# SEC61G Facilitates Brain Metastases via Antagonizing PGAM1 Ubiquitination and Immune Microenvironment Remodeling in Non-Small Cell Lung Cancer

**DOI:** 10.7150/ijbs.109187

**Published:** 2025-01-27

**Authors:** Changshuai Zhou, Yuechao Yang, Huanhuan Cui, Sen Li, Zhisu Wang, Lei Chen, Mingtao Feng, Deheng Li, Xin Chen, Bin Hao, Xiaojun Wu, Yang Gao, Liangdong Li, Jiayan Chen, Yiqun Cao

**Affiliations:** 1 Department of Neurosurgery, Fudan University Shanghai Cancer Center, Shanghai 200032, China.; 2 Department of Oncology, Shanghai Medical College, Fudan University, Shanghai 200032, China.; 3 Fudan University Shanghai Cancer Center and Institutes of Biomedical Sciences, Shanghai Medical College, Fudan University Shanghai Cancer Center, Shanghai 200032, China.; 4 Department of Radiation Oncology, Shanghai Medical College, Fudan University, Shanghai 200032, China.

**Keywords:** SEC61G, brain metastases, non-small cell lung cancer, metabolic reprogramming, PGAM1, ubiquitination, UBE3C, microglial polarization, tumor immune microenvironment

## Abstract

**Background:** Brain metastases are a leading cause of mortality in non-small cell lung cancer (NSCLC), yet their molecular mechanisms remain unclear. SEC61G, a subunit of the SEC61 translocon, has been implicated in tumor progression but its role in brain metastases is unknown. This study explores how SEC61G contributes to brain metastases by driving metabolic reprogramming and immune microenvironment remodeling.

**Methods:** Brain-metastatic NSCLC cell lines were established through *in vivo* selection in a mouse model. SEC61G expression was analyzed via transcriptomics, immunohistochemistry, multiplex immunofluorescence, and patient datasets. Functional assays were used to assess SEC61G's role in glycolysis, TLS formation, and immune interactions, with a focus on the SEC61G-PGAM1 axis. Pharmacological inhibitors and co-culture systems were employed to validate findings.

**Results:** SEC61G was identified as a key upregulated gene in brain metastases based on transcriptomic data from patient-derived samples and mouse models. Higher SEC61G expression in brain metastases correlated with advanced tumor stages and poor survival in NSCLC patients. Mechanistically, SEC61G promoted brain metastasis by stabilizing the key glycolytic enzyme PGAM1. This occurred through a novel mechanism of competitive inhibition of PGAM1 ubiquitination: SEC61G directly antagonized the E3 ubiquitin ligase UBE3C, preventing PGAM1 degradation via the proteasome pathway. Stabilized PGAM1 enhanced glycolysis and regulated oxidative phosphorylation, driving metabolic reprogramming that supported brain metastatic colonization. Moreover, SEC61G reshaped the tumor immune microenvironment by promoting microglial M2 polarization and suppressing M1 polarization, accompanied by increased secretion of IL-6 and IL-10. These immune effects were dependent on PGAM1, as its pharmacological inhibition reversed SEC61G-induced M2 polarization and restored CD8^+^ T cell infiltration. *In vivo* and clinical studies confirmed that high SEC61G expression in brain metastases correlated with excessive M2 microglia, reduced immune surveillance, and poor patient outcomes. Immunoprofiling revealed a striking gradient of SEC61G expression across tertiary lymphoid structures (TLS) maturation stages: SEC61G levels were highest in TLS-absent samples and CD206^+^ microglia infiltration, intermediate in samples with immature TLS, and lowest in those with mature TLS.

**Conclusion:** In conclusion, this study identifies a novel mechanism in which SEC61G drives NSCLC brain metastases by competitively inhibiting UBE3C-mediated ubiquitination of PGAM1, stabilizing PGAM1 and enhancing glycolysis. In addition to metabolic reprogramming, SEC61G impairs TLS maturation, suppresses adaptive immune responses, and facilitates immune evasion, contributing to brain metastatic colonization. These findings highlight SEC61G as a key regulator of brain metastasis and a promising therapeutic target for NSCLC patients with brain metastases.

## Introduction

Lung cancer is one of the leading causes of cancer-related mortality worldwide, with non-small cell lung cancer (NSCLC) accounting for approximately 85% of all lung cancer cases^1^. Despite significant progress in molecular-targeted therapies and immunotherapy in recent years, the overall prognosis for NSCLC patients remains poor^2^. Brain metastases are a common complication in NSCLC, occurring in approximately 40%-50% of patients with lung adenocarcinoma during disease progression^3^-^5^. This severe complication dramatically shortens survival, with a median survival time of less than one year upon occurrence. Brain metastases not only accelerate disease progression but also severely limit therapeutic options, as the blood-brain barrier (BBB) hinders the penetration of most drugs to reach effective concentrations in brain tissue^5^. Therefore, elucidating the molecular mechanisms underlying NSCLC brain metastases and identifying novel therapeutic targets are crucial to improving patient outcomes.

Tumor metastasis is a complex, multi-step process involving tumor cell invasion, survival in the circulatory system, adaptation to heterogeneous microenvironments, and eventual colonization and growth in distant tissues in NSCLC^6^. During brain metastasis, metabolic reprogramming and alterations in the tumor immune microenvironment have been identified as pivotal factors^7^, ^8^. To adapt to the unique brain microenvironment, tumor cells often enhance their glycolytic capacity, relying on glycolysis as the primary energy source even under aerobic conditions, a phenomenon known as the "Warburg effect"^9^, ^10^. Additionally, tumor cells remodel the immune microenvironment by inducing M2 polarization of brain-resident microglia, suppressing anti-tumor immune responses, and creating a supportive niche for survival and dissemination^11^. However, the specific mechanisms that regulate metabolic adaptation and immune microenvironment remodeling in NSCLC brain metastases remain poorly understood.

The SEC61 complex is a critical protein translocon in the endoplasmic reticulum (ER) that mediates the translocation of secreted and membrane proteins. Among its subunits, the gamma subunit (SEC61G) plays a key role in maintaining ER homeostasis and calcium balance^12^. Recent studies have revealed that SEC61G is aberrantly overexpressed in several solid tumors and promotes tumorigenesis and progression^13^. For instance, SEC61G has been found to enhance ER stress and activate the EGFR signaling pathway, promoting tumor growth and invasion in breast cancer^14^, ^15^. Similarly, SEC61G has been implicated in the progression of head and neck squamous cell carcinoma, colorectal, and renal cancer, where its overexpression is associated with increased tumor aggressiveness and drug resistance^12^, ^16^, ^17^. Notably, SEC61G has also been suggested to regulate tumor glycolysis, potentially playing a role in metabolic reprogramming that enables tumor cells to adapt to harsh microenvironments^15^. However, the specific role of SEC61G in NSCLC brain metastases and its underlying mechanisms remain unclear.

In addition to SEC61G, phosphoglycerate mutase 1 (PGAM1), a key enzyme in glycolysis, plays an essential role in tumor metabolic reprogramming^18^, ^19^. PGAM1 facilitates the conversion of glycolytic intermediates, providing energy and biosynthetic precursors for rapid tumor cell growth and proliferation. Studies have shown that PGAM1 is highly expressed in various cancers and is closely associated with tumor aggressiveness and metastasis^20^-^22^. For example, PGAM1 has been demonstrated to promote glycolysis and anti-apoptotic pathways in breast, prostate, and ovarian cancers^23^-^25^. Furthermore, PGAM1-mediated metabolic reprogramming can influence the tumor microenvironment, such as through lactate secretion, which modulates immune cell function and enhances immune evasion^26^. While PGAM1 has been implicated in NSCLC invasion and metastasis, its specific role in brain metastases remains to be fully elucidated^27^.

Of particular interest is the possibility that SEC61G and PGAM1 may function coordinately to regulate tumor metabolism and the immune microenvironment during brain metastasis^28^. For instance, SEC61G might regulate PGAM1 expression or activity, further enhancing glycolytic metabolism. Conversely, metabolic byproducts of PGAM1, such as lactate, may promote SEC61G-related pathways, collectively supporting tumor cell colonization and growth in the brain^20^, ^29^. Moreover, both SEC61G and PGAM1 may contribute to microglial polarization (M1/M2 states), remodeling the brain metastatic niche and facilitating immune evasion and tumor progression^30^.

Therefore, while SEC61G and PGAM1 have been implicated in promoting tumor progression in various cancers, their specific roles and interactions in NSCLC brain metastases remain unclear. This study aims to systematically investigate the biological functions of SEC61G in NSCLC brain metastases, with a particular focus on its potential regulation of PGAM1 activity and metabolic reprogramming, as well as its impact on the tumor immune microenvironment. By elucidating these mechanisms, this research seeks to provide novel insights into the pathogenesis of NSCLC brain metastases and identify potential therapeutic targets for improving treatment outcomes.

## Methods and Materials

### Cell lines and culture

The human non-small cell lung cancer (NSCLC) cell lines H2030, PC9, and their brain metastasis sublines H2030-BrM5 and PC9-BrM3 were obtained from the Chinese Academy of Sciences Cell Bank (Shanghai, China). H2030 and H2030-BrM5 cells were cultured in RPMI-1640 medium supplemented with 10% fetal bovine serum (FBS) and 1% penicillin-streptomycin. PC9 and PC9-BrM3 cells were cultured in Dulbecco's Modified Eagle Medium (DMEM) containing 10% FBS and 1% penicillin-streptomycin. All cells were maintained in a humidified incubator at 37°C with 5% CO_2_. Cells were routinely tested for mycoplasma contamination and authenticated using short tandem repeat profiling.

To generate H2030-BrM5 and H2030-BrM5-shSEC61G cell lines stably expressing luciferase, cells were transfected with a eukaryotic expression plasmid encoding the Firefly luciferase gene (e.g., pGL4.51 [luc2/CMV/Neo], Promega). Transfection was performed using Lipofectamine™ 3000 (Thermo Fisher Scientific) according to the manufacturer's protocol. After 48 hours, cells were subjected to selection with G418 (600 μg/mL) for 2-3 weeks to ensure stable integration of the luciferase gene. Clones resistant to G418 were expanded and tested for luciferase activity using a dual-luciferase reporter assay system (Promega) following the manufacturer's instructions. Briefly, 1 × 10⁴ cells were seeded into 96-well plates, lysed, and luciferase activity was quantified using a luminometer. Clones with consistent and robust luciferase expression were selected for further *in vivo* and *in vitro* studies.

### Animal models

Male BALB/c nude mice (4-week-old, SPF grade) were purchased from Shanghai Jihui Laboratory Animal Co., Ltd. 2-5×10^5^ (100 μl) luciferase-labeled cells H2030-BrM5-Luciferase and H2030-BrM5-shSEC61G-Luciferase were inoculated in 8 mice. For brain metastasis models, mice were injected with 2-5 x 10⁵ tumor cells via left ventricular injection. Animals were monitored for neurological symptoms and sacrificed at ethical endpoints. Tumor tissues were collected for histopathology and immunohistochemical (IHC) analysis. All animal experiments were approved by the Ethics Committee of Fudan University Cancer Hospital and conducted in accordance with institutional guidelines.

### Western blotting

Protein lysates were prepared using RIPA lysis buffer supplemented with protease and phosphatase inhibitors. Proteins were separated on 10% SDS-PAGE gels and transferred to PVDF membranes. Membranes were blocked with 5% skim milk in TBST for 1 hour at room temperature before incubation with primary antibodies overnight at 4°C. After washing, membranes were incubated with horseradish peroxidase (HRP)-conjugated secondary antibodies for 1 hour at room temperature. Signals were detected using enhanced chemiluminescence (ECL) reagents and quantified using ImageJ software.

### Quantitative PCR (qPCR)

Total RNA was extracted from cells using TRIzol reagent and reverse-transcribed into cDNA using PrimeScript™ RT Master Mix (Takara). Quantitative PCR was performed using TB Green™ Premix Ex Taq™ II (Takara) on a QuantStudio™ 7 Flex Real-Time PCR System. Relative gene expression was calculated using the 2⁻ΔΔCt method, with GAPDH as the internal control. Primer sequences are listed in **Table S1**.

### Immunohistochemistry (IHC)

Lung cancer primary and brain metastasis tissues were formalin-fixed, paraffin-embedded, and sectioned at 4 μm. Sections were deparaffinized, rehydrated, and subjected to antigen retrieval using citrate buffer (pH 6.0). After blocking with 5% bovine serum albumin (BSA), sections were incubated with primary antibodies at 4°C overnight, followed by HRP-conjugated secondary antibodies. Staining was visualized using DAB chromogen, counterstained with hematoxylin, and evaluated under a light microscope.

### Cell viability and proliferation assays

Cell viability was assessed using the Cell Counting Kit-8 (CCK-8) assay. Briefly, cells were seeded in 96-well plates at 2 × 10³ cells per well and treated as indicated. At specific time points, 10 μL of CCK-8 reagent was added to each well, and absorbance was measured at 450 nm using a microplate reader. For proliferation assays, cells were seeded in 6-well plates and counted daily using a hemocytometer.

### Seahorse glycolysis assays

Glycolytic function was assessed using a Seahorse XF Glycolysis Stress Test Kit on a Seahorse XFe96 Analyzer (Agilent Technologies). In brief, cells were seeded in Seahorse XF96 plates and allowed to adhere overnight. The assay measured extracellular acidification rate (ECAR) in response to glucose, oligomycin, and 2-deoxy-D-glucose (2-DG). Data were normalized to protein content per well, and glycolysis parameters were calculated using Wave software.

### Co-Immunoprecipitation (Co-IP)

Cells were lysed in IP lysis buffer containing protease and phosphatase inhibitors. Lysates were pre-cleared with protein A/G magnetic beads and incubated with primary antibodies or IgG controls overnight at 4°C. Immune complexes were captured using protein A/G beads, washed extensively, and analyzed by Western blotting. Input lysates and immunoprecipitated samples were probed for target proteins to confirm specific interactions.

### Plasmid construction and mutagenesis

To construct expression vectors for PGAM1-Flag, UBE3C-Myc, and HA-Ub mutants, coding sequences (CDS) of the target genes were retrieved from the NCBI database, with the longest transcript isoforms selected for cloning. For PGAM1 and UBE3C, primers were designed to facilitate insertion into the pCDH-CMV-MCS-EF1-Puro expression vector, incorporating Flag and Myc tags, respectively. The PGAM1 sequence was amplified using cDNA synthesized from H2030 cell RNA as a template, while UBE3C was amplified similarly. PCR amplification was performed using high-fidelity DNA polymerase, and the products were analyzed via agarose gel electrophoresis. The resulting fragments and vectors were digested with appropriate restriction enzymes (EcoRⅠ/BamHⅠ for PGAM1 and XbaⅠ/NotⅠ for UBE3C), purified, and ligated using T4 DNA ligase. The ligation products were transformed into DH5α competent cells, and positive clones were confirmed by PCR and Sanger sequencing. Verified plasmids were extracted using a plasmid purification kit and stored for subsequent experiments.

For the construction of HA-Ub mutants, site-directed mutagenesis was employed to replace the seven lysine residues (K6, K11, K27, K29, K33, K48, and K63) in ubiquitin with arginine (R), preventing ubiquitin chain formation. Mutation primers were designed to include the target lysine codon at the center, flanked by 9-15 bp of upstream and downstream sequences. Using the pCMV-HA-Ub plasmid as a template, mutagenesis was performed with a site-directed mutagenesis kit, followed by DpnI digestion to remove the parental plasmid. The mutated plasmids were transformed into competent cells, and positive clones were verified by sequencing. Plasmids with confirmed mutations were extracted and prepared for functional analysis.

### Tissue Microarray (TMA) transcriptome sequencing

A tissue microarray (TMA) containing 79 formalin-fixed, paraffin-embedded lung adenocarcinoma samples, along with matched adjacent normal tissues, was purchased from Shanghai Outdo Biotech Co., Ltd. Clinical data for these samples included patient age, gender, TNM staging, EGFR mutation status, lymph node metastasis status, distant metastasis, and overall survival (OS). Inclusion criteria required histopathological confirmation of adenocarcinoma, complete clinical data, and no prior chemotherapy or radiotherapy. SEC61G protein expression was measured by immunohistochemistry, and survival curves were generated based on SEC61G expression levels to assess its prognostic relevance.

All study design and experimental procedures for this study were carried out in accordance with Declaration of Helsinki II. Ethical approval and patient participation consent were approved and approved by the Ethics Committee of FUSCC. The postoperative specimens were officially identified as ccRCC through pathology examination. Tissue samples, including ccRCC and adjacent normal tissues, were collected during surgery and fixed in 4% paraformaldehyde; these were available from the FUSCC tissue bank.

### Co-culture system for lung cancer cells and microglia

A transwell-based co-culture system was established to model the interaction between lung cancer cells and microglia. Human lung cancer cell line H2030 (and its stable transfectants) was seeded in the upper chamber of a 6-well transwell plate (pore size: 0.4 μm), while the human microglial cell line HMC3 was cultured in the lower chamber. HMC3 cells were plated at an appropriate density in the lower chamber after trypsinization and allowed to adhere overnight to form a uniform monolayer. The next day, H2030 cells were seeded in the upper chamber. The two cell types were co-cultured for 72 hours, during which soluble factors in the shared medium facilitated paracrine communication, mimicking the tumor microenvironment. After co-culture, microglial cells were collected from the lower chamber and analyzed for phenotypic and functional changes using flow cytometry. This system provided a controlled platform to study tumor-microglia interactions.

### ELISA for cytokine quantification in co-culture supernatant

The impact of SEC61G and PGAM1 interaction on microglial polarization was assessed by measuring IL-6 and IL-10 levels in co-culture supernatants using ELISA. After 24 hours of co-culture under different conditions (Vector, SEC61G-OE, SEC61G+HKB99, and SEC61G-siPGAM1 groups), supernatants were collected and analyzed with ELISA kits according to the manufacturer's instructions. Cytokine concentrations were quantified using a microplate reader based on standard curves, and results were expressed as the mean ± S.E.M. of three independent experiments. Data were visualized in bar graphs to illustrate changes in cytokine levels associated with SEC61G and PGAM1 modulation.

### Bioinformatics analysis

The LUAD dataset from The Cancer Genome Atlas (TCGA) was utilized to evaluate the expression patterns, clinical relevance, and prognostic significance of SEC61G in lung adenocarcinoma (LUAD). SEC61G expression levels were analyzed in relation to clinical characteristics, including age (≥65 vs. <65), gender (male vs. female), pathological stage (Stage I-IV), clinical T stage (T1-T4), N stage (N0 vs. N1-3), M stage (M0 vs. M1), and survival status (alive vs. deceased). Gene expression data from the Gene Expression Omnibus (GEO) database were analyzed to validate SEC61G expression in lung adenocarcinoma and its association with brain metastasis. The datasets included GSE31210, GSE63459, GSE10072, GSE30219, and GSE19188, encompassing tumor and adjacent normal lung tissue samples. The raw data were normalized, ID-mapped, and cleaned before downstream analysis. SEC61G expression levels were correlated with clinical features and patient outcomes, and survival analyses were conducted to determine its prognostic significance.

### Single-cell RNA sequencing analysis

Single-cell RNA sequencing data from GSE131907 were analyzed to investigate SEC61G expression in primary lung adenocarcinoma tissues and brain metastases. The dataset included six primary tumor samples (e.g., P0009, P0018) and four brain metastasis samples (e.g., P3003, P3006). SEC61G expression was visualized across different cell populations using the URECA single-cell analysis platform. Cellular distribution and differential expression of SEC61G were compared between primary and metastatic sites.

Three lung adenocarcinoma brain metastasis samples from Fudan University Shanghai Cancer Center were analyzed using single-cell RNA sequencing on the 10x Genomics Chromium platform. Single-cell suspensions were prepared through enzymatic dissociation and filtration, with samples >85% viable confirmed by trypan blue staining. Libraries were constructed using the Chromium Single Cell 3' Reagent Kits (v3) and sequenced on the Illumina NovaSeq 6000 platform at 50,000 reads per cell. Data preprocessing, including alignment, feature-barcode matrix generation, and cell calling, was done using CellRanger (v6.0). The data were normalized and analyzed with Seurat (v4.0) in R, using PCA for dimensionality reduction and UMAP visualization. Tumor epithelial cells were clustered based on gene expression profiles. The SEC61G expression distribution was mapped across clusters, and SEC61G high and low expression clusters were compared to explore relevant pathways. Results were validated through independent replicates.

### Flow cytometry analysis of microglial polarization

A Transwell® system (0.4 µm pore size, Corning) was used to co-culture H2030 lung cancer cells and HMC3 microglial cells. HMC3 cells (5 × 10⁶) were seeded in the lower chamber and allowed to adhere overnight. The next day, 5 × 10⁶ H2030 cells were seeded in the upper chamber. After 24 hours of co-culture, 5 µM PGAM1 inhibitor was added to the culture medium of SEC61G-overexpressing H2030 cells. Microglial cells were collected from the lower chamber after 24 hours, washed with PBS, and divided for analysis.

Microglial cells were blocked with 1% BSA/PBS containing 0.5 μL Human TruStain FcX™ (BioLegend) at room temperature for 10 minutes. Cells were then stained with CD86-FITC and CD206-PE antibodies (1 μL each, diluted in 20 μL Perm Buffer) for 30 minutes, followed by two PBS washes. Stained cells were analyzed using flow cytometry to assess polarization states. CD86 and CD206 markers were used to identify M1 and M2 phenotypes, respectively. This approach provided insights into microglial responses under different experimental conditions.

### Statistical analysis

All experiments were performed in triplicate unless otherwise stated. Data are presented as mean ± standard deviation (SD). Statistical significance was determined using Student's t-test or one-way ANOVA followed by Tukey's post hoc test. Kaplan-Meier analysis was used for survival data, and log-rank tests were performed to compare groups. A p-value < 0.05 was considered statistically significant. Analyses were conducted using GraphPad Prism 9.0 software.

## Results

### Establishment of brain metastasis-susceptible lung cancer cell lines in mice

**Figure 1:**
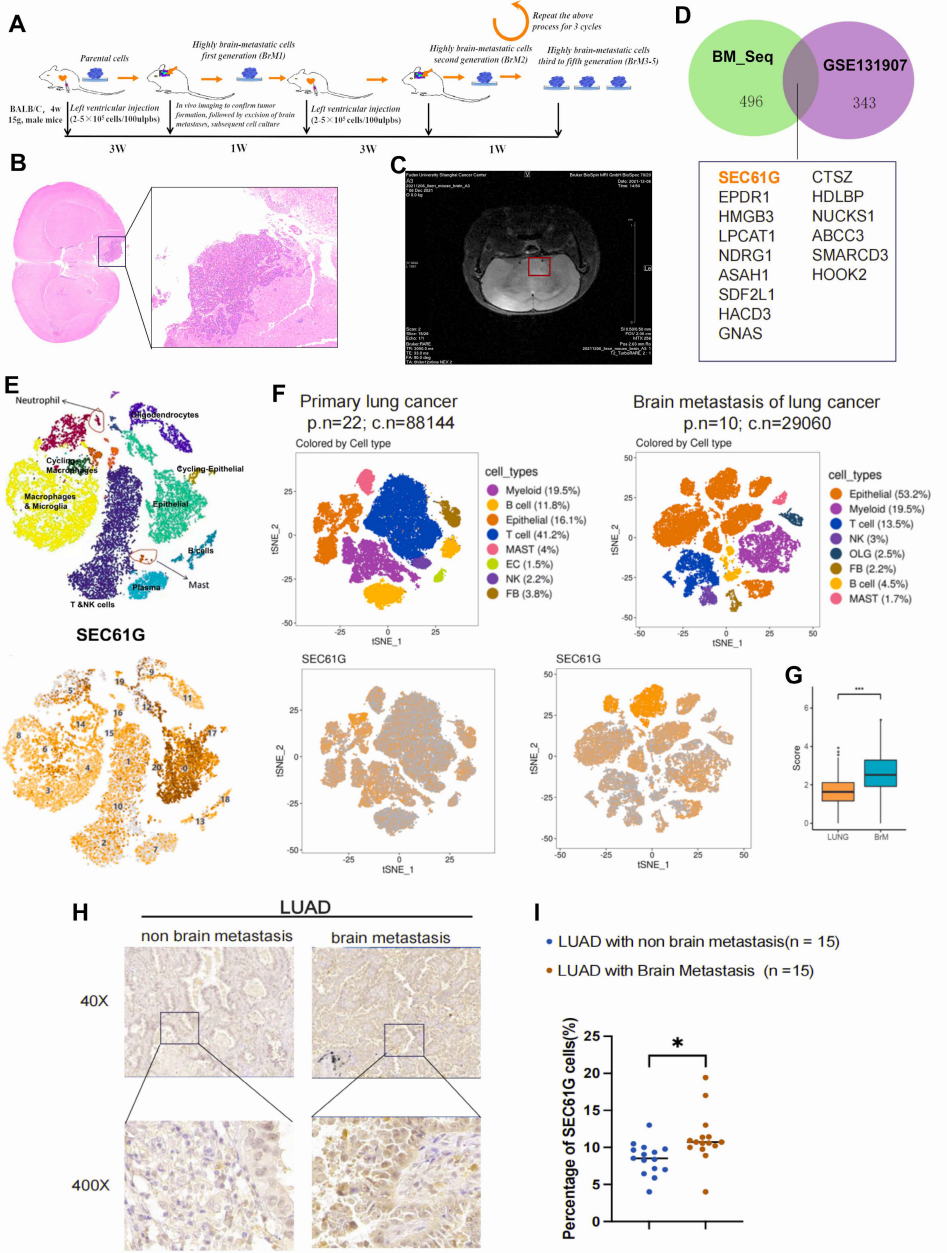
** Establishment of brain metastasis-susceptible lung cancer cell lines and identification of brain metastasis-associated genes in mice.** (A) Schematic representation of the experimental workflow for generating brain metastasis-susceptible lung cancer cell lines. H2030 and PC9 cells were labeled with luciferase and injected into the left ventricle of nude mice to mimic brain metastasis. (B) Representative hematoxylin-eosin (HE) staining of brain metastases from mice injected with H2030 and PC9 cells, confirming histopathological evidence of brain tumors. (C) *In vivo* bioluminescence imaging and magnetic resonance imaging (MRI) were used to detect brain metastases with high precision. (D) Venn diagram showing the intersection of differentially expressed genes identified from GSE131907 and transcriptome sequencing of H2030-BrM5 cells compared to parental H2030 cells, highlighting 15 overlapping genes, including SEC61G. (E) Single-cell RNA sequencing analysis of three brain metastasis samples revealed significant enrichment of SEC61G in tumor cell populations. (F) t-SNE analysis using the GSE131907 dataset demonstrated significant enrichment of SEC61G in tumor cells from brain metastases compared to primary lung cancer lesions. (G) Box plot illustrating the significantly elevated expression levels of SEC61G in brain metastatic tumor cells compared to primary tumor cells (P < 0.05). (H) Immunohistochemistry (IHC) analysis of SEC61G in clinical tissue samples (15 primary lung cancer tissues with brain metastases and 15 without brain metastases). Representative images show elevated SEC61G protein levels in brain metastatic lesions. (I) Quantification of IHC staining intensity confirmed significantly higher SEC61G expression in brain metastatic lesions compared to primary lung cancer tissues (P < 0.05).

In this study, H2030 and PC9 lung cancer cell lines were utilized as tumor models. After labeling these cells with a luciferase reporter gene, they were injected into the left ventricle of nude mice to mimic the process of brain metastasis (**Figure 1A**). To establish lung cancer cell lines with high brain metastatic potential, the mice were monitored for three weeks to assess their behavior and physical condition, providing preliminary evidence of tumor burden. Upon confirmation of tumor formation, a subset of mice was euthanized, and brain metastases were surgically excised for primary tumor cell culture. Brain tissues from other mice were subjected to fixation and hematoxylin-eosin (HE) staining for histological analysis (**Figure 1B**). Additionally, *in vivo* bioluminescence imaging and magnetic resonance imaging (MRI) were employed to confirm the presence of brain tumors with high precision (**Figure 1C**).

Using this approach, we successfully isolated primary lung cancer cells with high brain metastatic potential from the initial cohort of brain tumor-bearing mice, designated as H2030-BrM1 and PC9-BrM1. These cells were subsequently reinjected into the left ventricle of mice, inducing brain metastasis through repeated cycles of selection and propagation. Overall, we successfully developed lung cancer cell lines with high brain metastatic potential, providing robust models for studying the mechanisms underlying brain metastasis in lung cancer.

### Identification of brain metastasis-associated genes in mouse models

To uncover key genes associated with brain metastasis, two independent high-throughput sequencing datasets were analyzed. First, we examined the GSE131907 single-cell dataset from the GEO database. Differential expression analysis between epithelial cells from brain metastases and those from normal or primary lung adenocarcinoma tissues identified 343 differentially expressed genes. Second, transcriptome sequencing of the high brain metastatic cell line H2030-BrM5 (compared to its parental H2030 line) revealed 496 significantly differentially expressed genes (|log2 FC| > 1, P < 0.05), collectively referred to as the BM_seq gene set. Heatmaps were generated to visualize the top 50 upregulated genes in each dataset, highlighting the most significantly altered expression patterns (**Supplementary Figure 1A-B**).

To further refine our analysis, we performed an intersection analysis of the two gene sets, identifying 15 genes that were consistently upregulated in brain metastases. Among these, SEC61G-encoding the gamma subunit of the SEC61 translocon in the endoplasmic reticulum-was prominently enriched. A Venn diagram (**Figure 1D**) visually illustrates the intersection between the two datasets, highlighting SEC61G as a potential driver gene for brain metastasis. Thus, we identified SEC61G and 14 other genes as potential drivers of lung cancer brain metastasis, laying the groundwork for further mechanistic studies.

### SEC61G expression is significantly upregulated in brain metastases of lung cancer

To further validate the expression pattern and biological significance of SEC61G in brain metastases, we employed a multi-faceted analytical approach. First, single-cell RNA sequencing data from three brain metastasis samples provided by the Neurosurgery Department of Fudan University Cancer Hospital were analyzed. The results revealed that SEC61G was highly enriched in tumor cell populations within brain metastases across all three samples (**Figure 1E**). Next, we revisited the GSE131907 single-cell dataset to compare primary lung cancer lesions with brain metastases. Using t-distributed stochastic neighbor embedding (t-SNE) analysis, we observed significant enrichment of SEC61G in tumor cells from brain metastases (**Figure 1F**). Furthermore, the expression level of SEC61G was markedly elevated in brain metastatic tumor cells compared to primary tumor cells, with statistical significance (**Figure 1G**).

To substantiate these findings, we collected 15 primary lung cancer tissue samples with brain metastasis and 15 lung cancer tissues without brain metastases for immunohistochemical (IHC) analysis of SEC61G expression (**Figure 1H**). The results demonstrated that SEC61G protein levels were significantly higher in brain metastatic lesions than in primary lung cancer tissues (**Figure 1I**). Collectively, these results confirm that SEC61G is significantly up-regulated in lung cancer brain metastases, suggesting its potential role as a key regulator and biomarker of metastatic progression.

### SEC61G is overexpressed in LUAD and associated with poor survival

**Figure 2:**
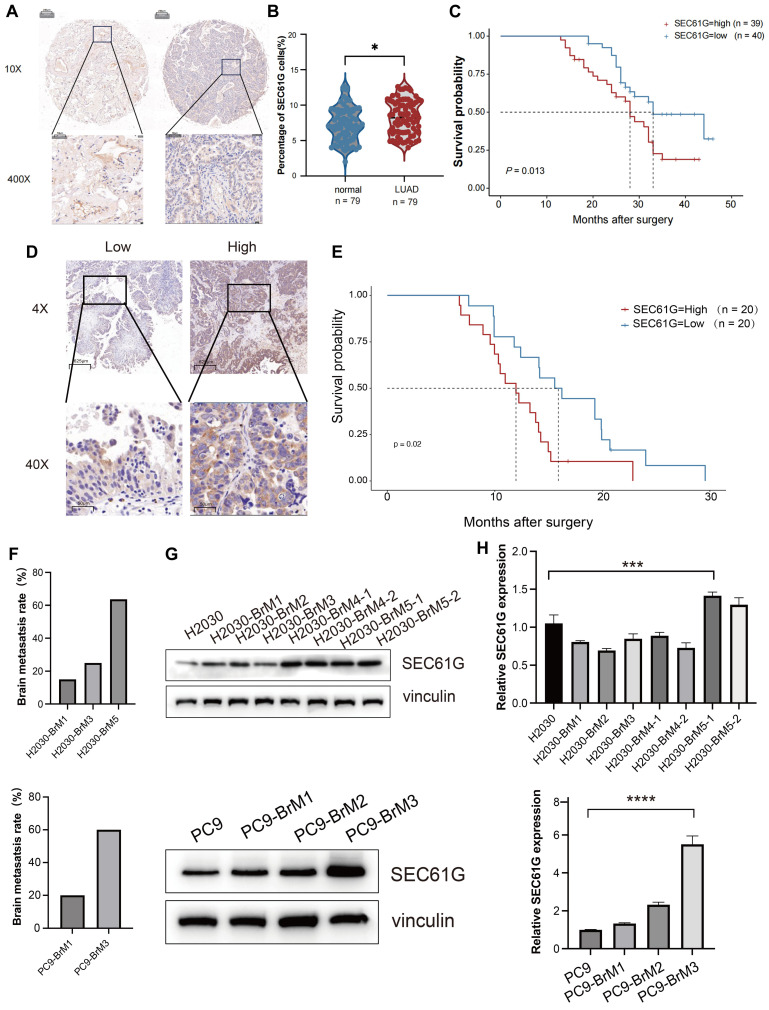
** SEC61G overexpression correlates with brain metastatic potential and poor survival in lung cancer.** (A) Representative immunohistochemical (IHC) staining of SEC61G in lung adenocarcinoma (LUAD) tissues and adjacent normal tissues, demonstrating significant upregulation in tumor tissues. (B-C) Kaplan-Meier survival curves showing shorter overall survival (OS) in LUAD patients with high SEC61G expression. (D) IHC staining of SEC61G in brain metastases from lung cancer patients, stratified into high and low expression groups based on immunoreactive scores. (E) Kaplan-Meier survival curves showing shorter survival in patients with high SEC61G expression in brain metastases. (F) Bar graph showing increased brain metastasis incidence in successive *in vivo* passages of H2030 and PC9 cells, correlating with increased SEC61G expression. (G-H) SEC61G mRNA and protein expression levels in brain-metastatic sublines (H2030-BrM1 to H2030-BrM5 and PC9-BrM1 to PC9-BrM3) compared to parental cell lines, demonstrating significant upregulation in brain-metastatic sublines.

To validate our earlier findings and further investigate the clinical significance of SEC61G in LUAD, we analyzed the protein expression of SEC61G in 79 patient samples collected from 2010 to 2015. These samples included both tumor tissues and adjacent normal tissues, and immunohistochemical (IHC) staining was used to assess SEC61G expression. The results demonstrated that SEC61G protein levels were significantly higher in LUAD tissues compared to adjacent normal tissues (**Figure 2A**). Moreover, SEC61G expression in tumor tissues showed a significant negative correlation with patient survival. Patients with higher SEC61G expression exhibited shorter overall survival, further emphasizing its potential role as a prognostic biomarker (**Figures 2B-C**).

To extend these findings, we performed IHC analysis on postoperative brain metastasis samples from 40 lung cancer patients with confirmed brain metastases. Based on immunoreactive scores, patients were divided into high and low SEC61G expression groups (**Figure 2D**). Survival analysis revealed that patients in the high SEC61G expression group had a median survival of 12.0 months, significantly shorter than the 15.9 months observed in the low SEC61G expression group (**Figure 2E**). This difference was statistically significant, indicating a strong association between high SEC61G expression and poor prognosis in lung cancer brain metastases.

To further investigate the prognostic value of SEC61G expression in lung adenocarcinoma (LUAD), we analyzed two independent cohorts: the TCGA-LUAD dataset (n = 535) and a LUAD tissue microarray cohort (n = 79). Based on the median expression level of SEC61G, patients in both cohorts were stratified into high and low expression groups. The association between SEC61G expression, clinical characteristics, and patient outcomes was then systematically analyzed.

In the LUAD tissue microarray cohort, high SEC61G protein expression was significantly associated with several adverse clinical characteristics, including advanced patient age (P = 0.013), T stage (P < 0.001), M stage (P < 0.001), and the number of lymph node metastases (P = 0.013) (**Table S2**). Furthermore, multivariate analysis demonstrated that high SEC61G expression was an independent predictor of poor prognosis in LUAD patients (P = 0.018) (**Table S3**).

Similarly, in the TCGA-LUAD dataset, high SEC61G mRNA expression was significantly correlated with lymph node metastasis (P = 0.023) and poor clinical outcomes, including OS (overall survival) events (P < 0.001), DSS (disease-specific survival) events (P = 0.006), and PFI (progression-free interval) events (P = 0.007) (**Table S4**). Multivariate analysis in this cohort confirmed that high SEC61G expression was an independent predictor of poor prognosis (P = 0.018) (**Table S5**).

In summary, SEC61G is significantly overexpressed in LUAD tissues and brain metastases, and its high expression is associated with shorter survival, emphasizing its potential as an independent prognostic biomarker in both primary and metastatic lung cancer.

### SEC61G expression increases with brain metastatic potential in lung cancer cell lines

Given the significant upregulation of SEC61G in lung cancer brain metastases, we further investigated its expression in lung cancer cell lines with varying brain metastatic potential. SEC61G expression increased progressively with successive *in vivo* passages, correlating with an increased incidence of brain metastasis in mice: H2030-BrM1 (18.2%, 2/11), H2030-BrM3 (27.2%, 3/11), H2030-BrM5 (63.4%, 7/11); PC9-BrM1 (20%, 2/10), PC9-BrM3 (60%, 6/10) (**Figure 2F**). Based on these findings, we selected the fifth-generation high brain metastatic cell line H2030-BrM5 and the third-generation high brain metastatic cell line PC9-BrM3 as representative models for subsequent experiments.

Moreover, using a series of brain-metastatic sublines (H2030-BrM1 to H2030-BrM5 and PC9-BrM1 to PC9-BrM3) and their corresponding parental lines (H2030 and PC9), we analyzed SEC61G expression at both the transcriptomic and proteomic levels. The results revealed that SEC61G mRNA and protein levels were significantly elevated in brain-metastatic cell lines compared to their parental cell lines (**Figures 2G-H**).

These results suggest a strong correlation between SEC61G expression and the brain metastatic potential of lung cancer cells. Although these findings highlight SEC61G as a potential molecular marker and mediator of brain metastasis, additional studies are required to determine whether SEC61G acts as a driver gene in lung cancer brain metastasis.

### SEC61G overexpression activates epithelial-mesenchymal transition and glycolysis pathways in lung cancer cells

**Figure 3:**
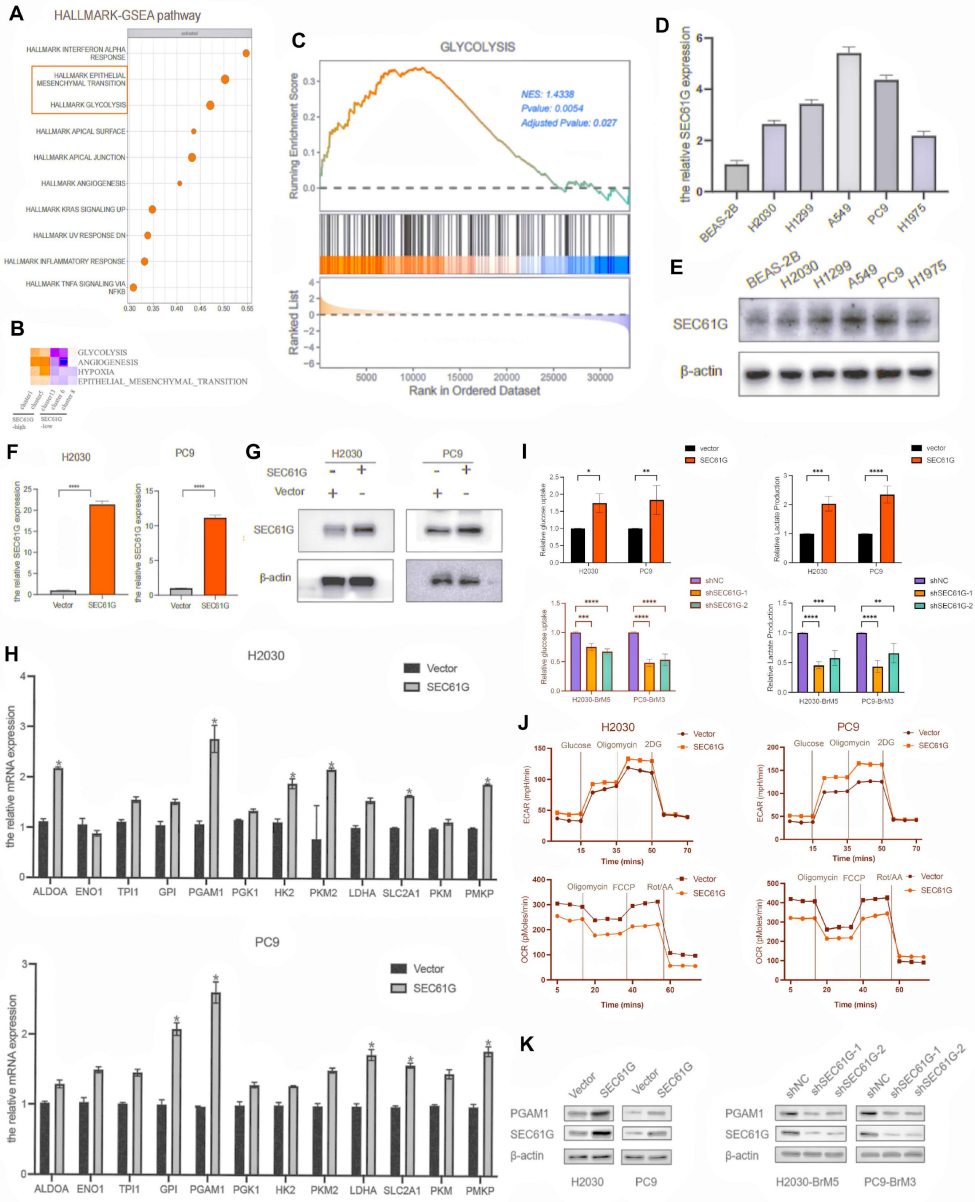
** SEC61G overexpression activates Epithelial-Mesenchymal Transition (EMT) and glycolysis pathways in lung cancer cells.** (A) RNA bulk transcriptome sequencing of SEC61G-overexpressing H2030 cells reveals activation of EMT and glycolysis pathways. (B) Hallmark pathway enrichment analysis of single-cell RNA sequencing data from brain metastases shows significant enrichment of glycolysis and EMT pathways in cells with high SEC61G expression. (C) Gene set enrichment analysis (GSEA) of glycolysis-related genes demonstrates significant activation of glycolysis in SEC61G-overexpressing cells (normalized enrichment score [NES] = 1.43). (D-E) Western blot showing low baseline SEC61G expression in H2030 and PC9 cells compared to other NSCLC cell lines. (F-G) Validation of SEC61G overexpression in stable H2030 and PC9 cell lines. (H) RT-PCR showing upregulation of glycolytic enzyme genes, particularly PGAM1, in SEC61G-overexpressing cells. (I) Glucose uptake and lactate production assays reveal increased glycolytic activity in SEC61G-overexpressing cells and reduced activity upon SEC61G knockdown. (J) Seahorse XFe analysis showing increased extracellular acidification rate (ECAR) and decreased oxygen consumption rate (OCR) in SEC61G-overexpressing cells, indicating a shift toward aerobic glycolysis. (K) Western blot showing increased PGAM1 protein levels in SEC61G-overexpressing cells and decreased levels upon SEC61G knockdown.

To investigate the biological mechanisms by which SEC61G affects tumor phenotypes, RNA bulk transcriptome sequencing was performed on H2030 cells overexpressing SEC61G. The results revealed that SEC61G overexpression was associated with the activation of key biological processes, including epithelial-mesenchymal transition (EMT) and glycolysis (**Figure 3A**). These findings were further supported by single-cell sequencing data from brain metastases, which demonstrated that cells with high SEC61G expression exhibited significant enrichment of glycolysis and EMT pathways, as determined by Hallmark pathway enrichment analysis (**Figure 3B**).

To further validate these findings, a gene set enrichment analysis (GSEA) of glycolysis-related genes was conducted. The analysis showed that the glycolysis pathway was significantly activated in cells with high SEC61G expression, with a normalized enrichment score (NES) of 1.43 (P < 0.01, **Figure 3C**). These results suggest that SEC61G plays a pivotal role in the metabolic reprogramming of lung cancer cells, particularly in enhancing glycolysis, which provides a foundation for further mechanistic studies.

### SEC61G promotes glycolysis by regulating the expression of key metabolic enzymes

To explore the potential metabolic effects of SEC61G, we first examined its mRNA and protein expression in normal lung epithelial cells and various NSCLC cell lines. Western blot analysis revealed that SEC61G was expressed at relatively low levels in H2030 and PC9 cells, which were selected for further studies (**Figures 3D-E**). Using these cell lines, stable SEC61G-overexpressing cell lines were successfully constructed and validated (**Figures 3F-G**).

As glycolysis is a series of tightly regulated reactions mediated by key metabolic enzymes, we conducted RT-PCR to screen for changes in glycolysis-related enzyme expression in SEC61G-overexpressing H2030 and PC9 cells. The results showed that SEC61G overexpression led to significant changes in the expression of several glycolytic enzymes, with the most prominent being the upregulation of Phosphoglycerate Mutase 1 (PGAM1). This pattern of PGAM1 upregulation was consistently observed across both cell lines (**Figure 3H**). CCK-8 assay indicated that upregulated SEC61G expression markedly increased proliferation ability of H2030 and PC9 cells (**Supplementary Figure 2**).

To confirm the functional impact of SEC61G on glycolysis, we assessed glucose uptake and lactate production in lung cancer cells. Overexpression of SEC61G in H2030 and PC9 cells significantly enhanced glucose uptake and lactate production (**Figure 3I**). Conversely, silencing SEC61G in the highly metastatic H2030-BrM5 and PC9-BrM3 cells markedly reduced glucose uptake and lactate production (Figure 3I). These results provide strong evidence that SEC61G promotes glycolysis in lung cancer cells.

### SEC61G overexpression enhances aerobic glycolysis and regulates oxidative phosphorylation in lung cancer cells

To further investigate how SEC61G influences lung cancer cell metabolism, we used Seahorse XFe analyzers to evaluate its effects on glycolytic capacity. The extracellular acidification rate (ECAR), an indicator of glycolytic activity, and the production of carbon dioxide (CO_2_) during mitochondrial respiration, a marker of oxidative phosphorylation, were measured. The results demonstrated that SEC61G overexpression significantly increased the level of aerobic glycolysis, as evidenced by elevated ECAR, while concurrently regulating oxidative phosphorylation (**Figure 3J**). This metabolic shift highlights SEC61G's role in promoting tumor cell dependency on glycolysis over mitochondrial respiration.

To validate these findings, Western blot experiments were conducted to assess the protein levels of PGAM1 in SEC61G-overexpressing and SEC61G-silenced lung cancer cells. The results showed that SEC61G overexpression significantly increased PGAM1 protein levels, while silencing SEC61G led to a marked decrease in PGAM1 expression in both H2030 and PC9 cells (**Figure 3K**). These findings suggest that SEC61G may regulate glycolysis by directly or indirectly modulating PGAM1 expression, thereby promoting metabolic reprogramming in lung cancer cells.

### SEC61G physically interacts with PGAM1 in lung cancer cells

**Figure 4:**
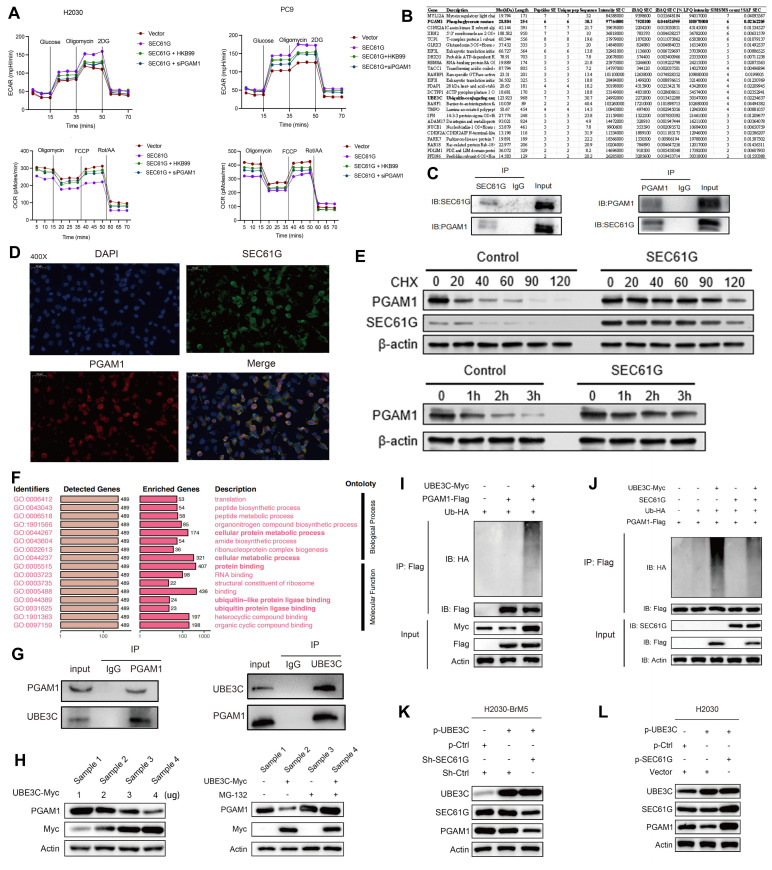
** SEC61G physically interacts with PGAM1 and enhances its stability by inhibiting ubiquitin-proteasome degradation.** (A) Extracellular acidification rate (ECAR) and oxygen consumption rate (OCR) measurements demonstrate that PGAM1 silencing or inhibition reverses SEC61G-induced glycolytic activation. (B) Immunoprecipitation followed by LC-MS identifies PGAM1 as a SEC61G-binding protein. (C) Co-immunoprecipitation (Co-IP) confirms endogenous interaction between SEC61G and PGAM1. (D) Immunofluorescence staining shows cytoplasmic co-localization of SEC61G and PGAM1 in H2030-BrM5 cells. (E) Cycloheximide (CHX) chase assay showing enhanced stability of PGAM1 in SEC61G-overexpressing cells. (F) Enrichment analysis reveals SEC61G-associated pathways related to metabolic regulation and ubiquitination. (G) Co-IP confirms endogenous interaction between UBE3C and PGAM1. (H) Western blot showing that UBE3C overexpression promotes PGAM1 degradation, which is inhibited by MG132 treatment. (I) Ubiquitination assay reveals UBE3C-mediated polyubiquitination of PGAM1. (J) Ubiquitination assay showing that SEC61G inhibits UBE3C-mediated ubiquitination of PGAM1. (K-L) Western blot showing knockdown of SEC61G expression decreases PGAM1 protein levels and counteracts UBE3C-induced PGAM1 degradation in H2030-BrM5 cells, while in H2030 cells, overexpression of SEC61G significantly increased PGAM1 levels.

In the glycolysis pathway, PGAM1 plays a critical role by converting 3-phosphoglycerate (3-PG) to 2-phosphoglycerate (2-PG), thereby accelerating glucose metabolism and promoting energy production. To elucidate the relationship between SEC61G and PGAM1 in regulating glycolysis, we used H2030 cells overexpressing SEC61G as a model. PGAM1 expression was suppressed using siRNA, or its enzymatic activity was inhibited with the small molecule inhibitor HKB99. The effects of these treatments on extracellular acidification rate (ECAR) and oxygen consumption rate (OCR) were then assessed. The results showed that overexpression of SEC61G alone significantly enhanced aerobic glycolysis (increased ECAR) and suppressed oxidative phosphorylation (decreased OCR). However, silencing PGAM1 or treating cells with HKB99 completely reversed these effects (**Figure 4A**). These findings indicate that SEC61G's promotion of glycolysis depends on the proper expression and activity of PGAM1.

To explore the molecular mechanisms underlying the relationship between SEC61G and PGAM1, we utilized immunoprecipitation (IP) combined with mass spectrometry (LC-MS) to identify SEC61G-binding proteins in H2030 cells stably overexpressing SEC61G. Interestingly, PGAM1 was identified as a candidate SEC61G-interacting protein (**Figure 4B**). This interaction was further validated using co-immunoprecipitation (Co-IP) experiments, which confirmed that SEC61G and PGAM1 interact endogenously (**Figure 4C**).

To visualize the subcellular localization of SEC61G and PGAM1, immunofluorescence staining was performed in H2030-BrM5 cells. SEC61G was labeled with red fluorescence, PGAM1 with green fluorescence, and the nucleus with DAPI. The results showed that SEC61G and PGAM1 co-localize in the cytoplasm, with overlapping fluorescence signals (yellow) observed in nuclear-adjacent regions (**Figure 4D**). In summary, SEC61G physically interacts with PGAM1 in lung cancer cells, and both proteins exhibit cytoplasmic co-localization, supporting their functional association.

### SEC61G stabilizes PGAM1 by inhibiting its ubiquitin-proteasome degradation

To investigate whether SEC61G regulates PGAM1 at the protein level, we assessed the stability of PGAM1 in SEC61G-overexpressing lung cancer cells using the protein synthesis inhibitor cycloheximide (CHX). H2030-vector and H2030-SEC61G cells were treated with CHX (100 μg/ml) at various time points (0, 20, 40, 60, 90, and 120 minutes), and PGAM1 protein levels were analyzed. In the control H2030-vector cells, PGAM1 protein levels gradually decreased over time, reflecting natural protein degradation (**Figure 4E**). However, in H2030-SEC61G cells, PGAM1 protein levels increased over time, suggesting that SEC61G overexpression enhances PGAM1 stability by activating a protective mechanism. To further confirm this, cells were treated with the proteasome inhibitor MG132 (**Figure 4E**). The results showed that PGAM1 degradation was significantly inhibited in SEC61G-overexpressing cells, indicating that SEC61G stabilizes PGAM1 by preventing its proteasomal degradation.

### SEC61G regulates ubiquitination processes and may interact with UBE3C

To further explore the signaling pathways associated with SEC61G in LUAD, enrichment analyses were performed using the BEST-LUAD public database. The results revealed that SEC61G-related pathways were predominantly involved in metabolic regulation and ubiquitination processes (**Figure 4F**). Transcriptomic analysis of lung cancer cell lines also showed a significant inverse correlation between SEC61G expression and ubiquitination-related biological processes (**Supplementary Figure 3A**).

Mass spectrometry experiments identified a potential interaction between SEC61G and the E3 ubiquitin ligase UBE3C (**Figure 4B**). UBE3C is a critical enzyme that catalyzes the covalent attachment of ubiquitin to target proteins, regulating their function and degradation. Given the previously observed stabilization of PGAM1 by SEC61G, it is possible that SEC61G modulates UBE3C activity or its interaction with PGAM1. Supporting this hypothesis, protein-protein interaction networks and epigenetic regulatory analyses revealed that SEC61G, PGAM1, and UBE3C are closely linked in processes such as protein ubiquitination and tumor metabolic regulation (**Supplementary Figure 2B**). Together, SEC61G may regulate PGAM1 stability and activity by modulating UBE3C-mediated ubiquitination, providing new insights into the role of SEC61G in tumor metabolic reprogramming.

### UBE3C Promotes PGAM1 Degradation via the Proteasome Pathway

Based on mass spectrometry and bioinformatics analyses, we hypothesized that UBE3C might mediate the effect of SEC61G on PGAM1 stability. To test this, we first used Co-IP to examine whether UBE3C and PGAM1 interact under endogenous conditions. The results confirmed that UBE3C and PGAM1 physically interact (**Figure 4G**). Next, we co-transfected HEK293T cells with increasing concentrations of UBE3C-Myc plasmid and a constant amount of PGAM1-Flag plasmid, followed by Western blot to detect PGAM1 protein levels. The results showed that UBE3C overexpression resulted in a dose-dependent decrease in PGAM1 protein levels (**Figure 4H**), suggesting that UBE3C promotes PGAM1 degradation.

To determine whether UBE3C-mediated PGAM1 degradation occurs via the proteasome pathway, cells were treated with the proteasome inhibitor MG132. This treatment significantly inhibited the UBE3C-induced degradation of PGAM1 (**Figure 4H**), confirming that UBE3C promotes PGAM1 degradation through a proteasome-dependent mechanism.

Protein ubiquitination is a major mechanism driving proteasome-dependent protein degradation. To investigate whether UBE3C promotes PGAM1 ubiquitination, HEK293T cells were co-transfected with UBE3C-Myc, PGAM1-Flag, and HA-Ub plasmids in various combinations. Ubiquitination assays revealed that UBE3C overexpression significantly increased the ubiquitination of PGAM1 (**Figure 4I**). These findings indicate that UBE3C acts as an E3 ubiquitin ligase, targeting PGAM1 for ubiquitination and subsequent degradation.

### SEC61G antagonizes UBE3C-mediated PGAM1 ubiquitination and promotes PGAM1 stability

Given that SEC61G binds to PGAM1 and enhances its stability, we hypothesized that SEC61G might antagonize the effect of UBE3C on PGAM1 degradation. To test this, HEK293T cells were co-transfected with UBE3C-Myc, PGAM1-Flag, SEC61G, and HA-Ub plasmids in various combinations. Ubiquitination assays showed that co-expression of SEC61G significantly suppressed UBE3C-mediated ubiquitination of PGAM1 (**Figure 4J**).

To further assess the functional interplay between SEC61G and UBE3C, we examined the effects of SEC61G expression changes on PGAM1 levels in lung cancer cell lines (**Figure 4K-L**). In H2030-BrM5 cells, knockdown of SEC61G reduced PGAM1 protein levels, while in H2030 cells, overexpression of SEC61G significantly increased PGAM1 levels. Moreover, the suppressive effect of UBE3C on PGAM1 protein levels was markedly attenuated in cells with high SEC61G expression. These results suggest that SEC61G stabilizes PGAM1 by antagonizing UBE3C-mediated ubiquitination, thereby reducing PGAM1 protein degradation.

### UBE3C promotes K48-linked ubiquitination of PGAM1

To investigate the specific type of ubiquitination modification mediating UBE3C-induced PGAM1 degradation, we utilized Ub-K0, a mutant form of ubiquitin in which all seven lysine residues (K6, K11, K27, K29, K33, K48, K63) were replaced with arginine (R), thereby preventing the formation of polyubiquitin chains. Additionally, we analyzed predicted ubiquitination sites on PGAM1 (amino acids 100-200) from the PhosphoSitePlus database, identifying five potential lysine ubiquitination sites (**Supplementary Figure 4**).

**Figure 5:**
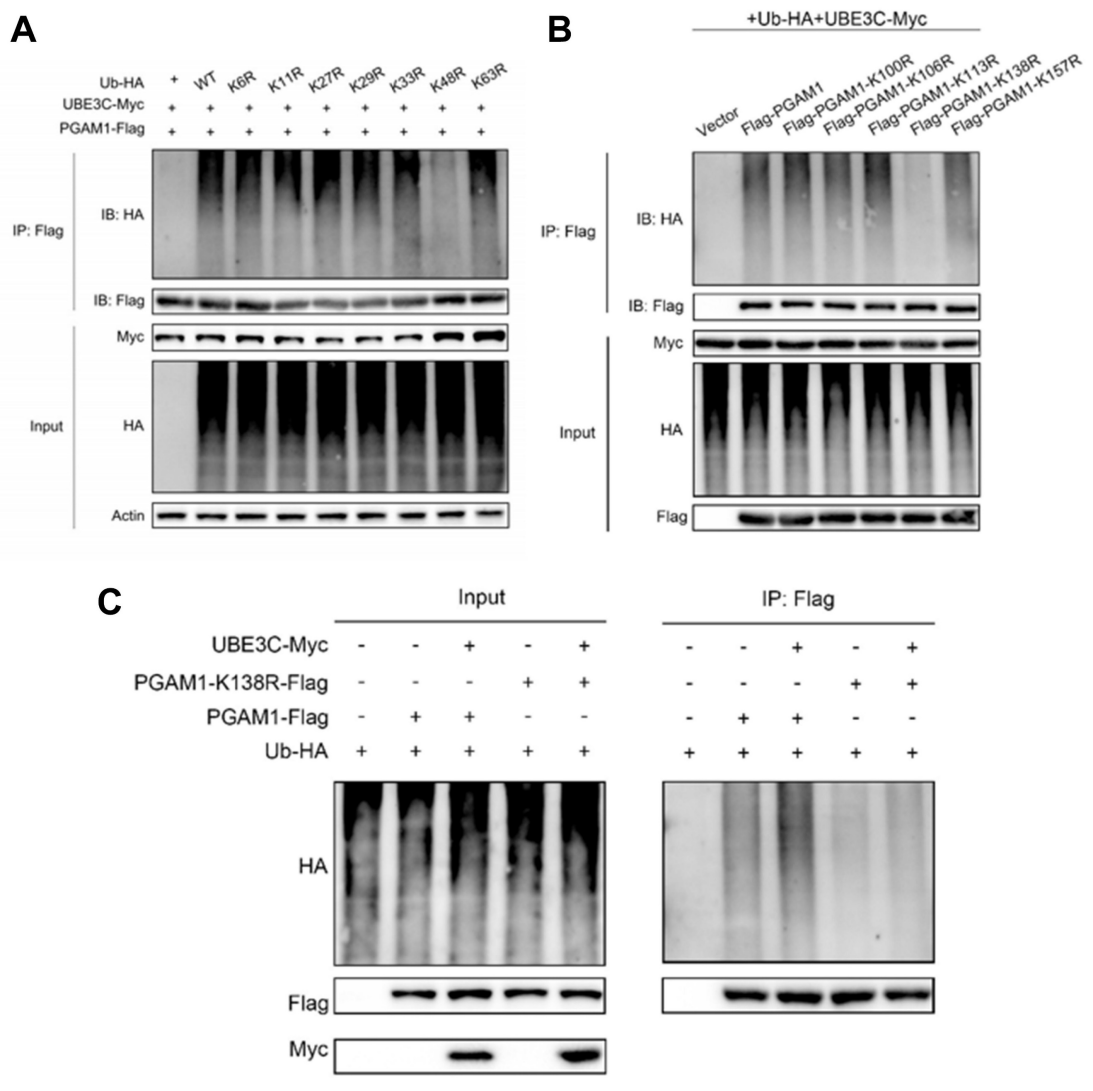
** SEC61G protects PGAM1 stability by blocking UBE3C-mediated ubiquitination at lysine 138 (K138).** (A) Schematic representation of lysine-to-arginine (K-to-R) mutants of PGAM1. Individual lysine residues (K100, K106, K113, K138, and K157) were replaced by arginine (R) to identify the specific site targeted by UBE3C for ubiquitination. (B) Ubiquitination assay performed in HEK293T cells transfected with Flag-PGAM1 wild-type (WT) or lysine mutants (K100R, K106R, K113R, K138R, and K157R) demonstrated that lysine 138 (K138) is the critical ubiquitination site on PGAM1 targeted by UBE3C. Mutation of K138 (K138R) significantly reduced PGAM1 ubiquitination compared to WT and other mutants. (C) SEC61G inhibits UBE3C-mediated ubiquitination of PGAM1 at lysine 138. Co-transfection of HEK293T cells with Myc-UBE3C, Flag-PGAM1, SEC61G, and HA-Ub revealed that SEC61G significantly reduces UBE3C-induced ubiquitination of PGAM1.

Using various HA-Ub mutants (Ub-K6R, Ub-K11R, Ub-K27R, Ub-K29R, Ub-K33R, Ub-K48R, Ub-K63R), we co-transfected Myc-UBE3C, Flag-PGAM1, and these ubiquitin mutants into HEK293T cells to assess the ubiquitination pattern of PGAM1. The results showed that PGAM1 ubiquitination levels were comparable to wild-type HA-Ub for most mutants, except for Ub-K48R. Co-transfection of Myc-UBE3C, Flag-PGAM1, and Ub-K48R significantly reduced PGAM1 ubiquitination (**Figure 5A**). This finding indicates that UBE3C mediates K48-linked polyubiquitination of PGAM1, a ubiquitin chain type associated with proteasome-dependent degradation.

### SEC61G protects PGAM1 stability by blocking UBE3C-mediated ubiquitination at lysine 138

To identify the specific site on PGAM1 targeted by UBE3C, we generated lysine mutants (K100R, K106R, K113R, K138R, K157R) where individual lysine residues were replaced with arginine (R). Ubiquitination assays revealed that K138 is the critical ubiquitination site on PGAM1 targeted by UBE3C, as mutation of this residue (K138R) caused a significant reduction in PGAM1 ubiquitination compared to wild-type PGAM1 or other mutants (**Figure 5B**). Further validation in H2030 cells confirmed that UBE3C-mediated ubiquitination of PGAM1 depends on lysine 138 (K138) (**Figure 5C**).

To test whether SEC61G antagonizes UBE3C at this specific site, we co-transfected HEK293T cells with Myc-UBE3C, Flag-PGAM1, SEC61G, and HA-Ub in various combinations. SEC61G significantly reduced UBE3C-mediated ubiquitination of PGAM1, suggesting that SEC61G competitively blocks UBE3C's access to the K138 site (**Figure 5C**). In the K138 mutant (K138R), UBE3C-induced ubiquitination was abolished, even in the absence of SEC61G, confirming that SEC61G protects PGAM1 stability by targeting the K138 site and preventing its ubiquitination. Collectively, SEC61G protects PGAM1 stability by antagonizing UBE3C-mediated ubiquitination at lysine 138 (K138), thereby preventing its degradation.

### SEC61G promotes M2 polarization of microglia while suppressing M1 polarization

**Figure 6:**
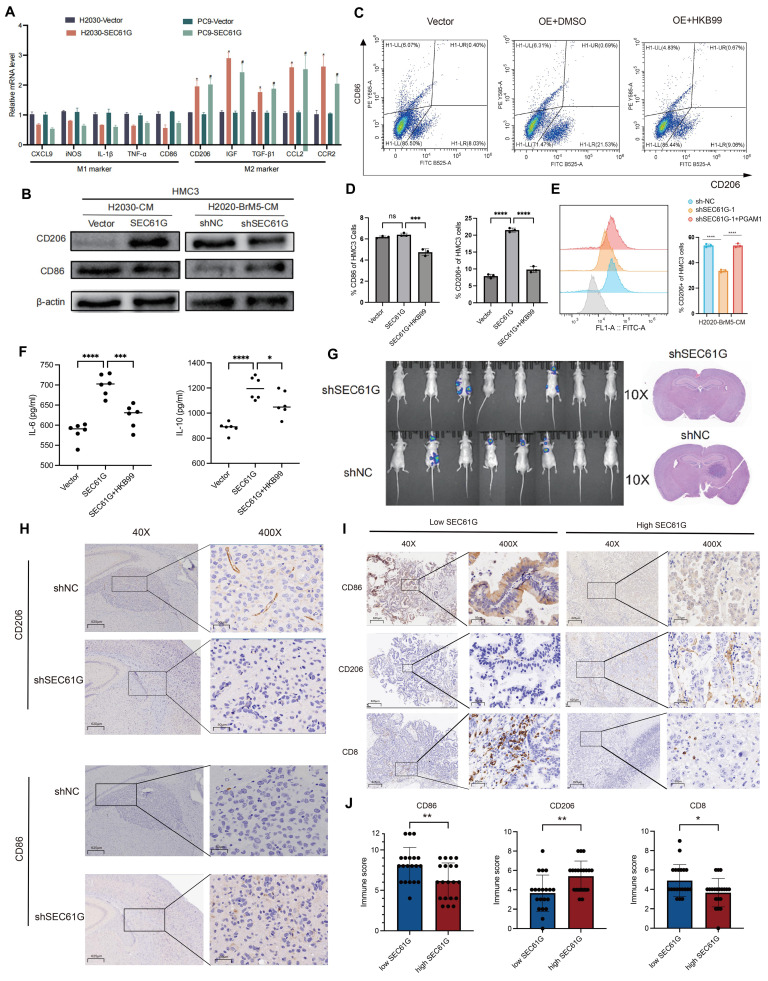
** SEC61G promotes M2 polarization of microglia and suppresses T cell infiltration in brain metastases.** (A) qPCR analysis showing markedly increased M2 marker expression and relatively reduced M1 marker expression in microglia co-cultured with SEC61G-overexpressing lung cancer cells. (B) Western blot analysis of CD206 (M2 marker) and CD86 (M1 marker) in microglia co-cultured with SEC61G-overexpressing or SEC61G-knockdown cells. (C) Flow cytometry analysis reveals increased CD206 expression in microglia co-cultured with SEC61G-overexpressing cells. (D) Addition of the PGAM1 inhibitor HKB99 reverses SEC61G-induced M2 polarization. (E) Conditioned media experiments confirm that PGAM1 overexpression rescues M2 polarization in SEC61G-knockdown cells. (F) ELISA results showing increased IL-6 and IL-10 secretion in co-cultures with SEC61G-overexpressing cells. (G) Representative bioluminescence imaging of brain metastases in nude mice injected with H2030-BrM5 or H2030-BrM5-shSEC61G cells. (H) IHC staining of CD206 (M2 marker) and CD86 (M1 marker) in brain sections from tumor-bearing mice. (I) IHC staining of CD206, CD86, and CD8 in clinical brain metastasis samples with high or low SEC61G expression. (J) Quantification of IHC staining scores shows that high SEC61G expression correlates with increased M2 polarization and reduced T cell infiltration, whereas low SEC61G expression correlates with increased M1 polarization and enhanced T cell infiltration.

To explore the impact of SEC61G on M1 and M2 polarization markers in microglia, we overexpressed SEC61G in H2030 and PC9 lung adenocarcinoma cells and co-cultured these cells with HMC3 microglia. Using qPCR, we quantified the mRNA expression of M1- and M2-specific markers in microglia (**Figure 6A**). The results revealed that SEC61G overexpression significantly reduced the mRNA levels of M1 polarization markers, while increasing the mRNA levels of M2 polarization markers in microglia. To further confirm the effects of SEC61G on microglial polarization, we manipulated SEC61G expression in lung cancer cells and co-cultured them with HMC3 microglia. In SEC61G-overexpressing H2030 cells, microglial CD206 (M2 marker) protein expression was significantly upregulated, while CD86 (M1 marker) protein expression was downregulated (**Figure 6B**). Conversely, in SEC61G-knockdown H2030-BrM5 cells, CD206 expression in microglia was reduced, and CD86 expression was increased (**Figure 6B**). Overall, SEC61G expression in lung cancer cells significantly influences the immune phenotype of microglia, promoting M2 polarization while inhibiting M1 polarization at the protein level.

To evaluate the effects of SEC61G on microglial polarization more precisely, we infected H2030 and PC9 cells with either an empty vector or a SEC61G-overexpression plasmid. After co-culturing with HMC3 microglia, we used flow cytometry to measure the expression of CD206 (M2 marker) and CD86 (M1 marker) on the microglial surface. Compared to the control group, SEC61G overexpression increased CD206 expression without affecting CD86 levels (**Figure 6C**). However, when the PGAM1 inhibitor HKB99 was added to the SEC61G-overexpressing system, CD206 expression decreased, and CD86 expression increased (**Figure 6D**). This indicates that PGAM1 is critical for SEC61G's ability to promote M2 polarization of microglia.

Recent studies have highlighted the role of these cytokines in fostering an immunosuppressive TME. TGF-β inhibits the activation and proliferation of CD8+ cytotoxic T cells, while IL-6 has been shown to activate STAT3 signaling, enhancing the pro-tumorigenic functions of M2 macrophages^31^, ^32^. To further confirm the role of SEC61G and PGAM1 in microglial polarization, we cultured HMC3 microglia with conditioned media (CM) from SEC61G-knockdown H2030-BrM5 cells. The results showed that CM from SEC61G-knockdown cells significantly reduced CD206 expression in microglia. However, when PGAM1 was transiently overexpressed in SEC61G-knockdown H2030-BrM5 cells, the conditioned media restored CD206 expression in microglia (**Figure 6E**). To investigate the effects of SEC61G on cytokine secretion, we co-cultured SEC61G-overexpressing H2030 cells with HMC3 microglia and measured the levels of IL-6 and IL-10 in the culture medium using ELISA. The results showed that, compared to the control group (Vector), the SEC61G-overexpressing group displayed significantly increased levels of IL-6 and IL-10 in the co-culture medium (**Figure 6F**). These findings suggest that PGAM1 overexpression can rescue the reduction in M2 polarization caused by SEC61G knockdown in lung cancer cells, highlighting the SEC61G-PGAM1 axis in regulating microglial polarization and the tumor immune microenvironment.

### SEC61G expression promotes M2 microglial polarization and suppresses T cell infiltration in brain metastasis

To examine the relationship between SEC61G expression and microglial polarization and temporal dynamics of microglial reprogramming in response to SEC61G modulation, we conducted animal experiments using luciferase-labeled H2030-BrM5 cells and shSEC61G-knockdown H2030-BrM5 cells (H2030-BrM5-shSEC61G) (**Figure 6G**). Tumor-bearing mouse brain tissues were fixed and sectioned for IHC staining of CD206 (M2 marker) and CD86 (M1 marker). The results showed that CD206 staining was weaker in the shSEC61G group, indicating reduced M2 polarization, while CD86 staining was stronger, indicating increased M1 polarization compared to the control group (**Figure 6H**). These results highlighting metabolic reprogramming orchestrates TAM plasticity in response to tumor-derived signals in lung cancer brain metastases.

To validate the findings from animal models, we analyzed 40 clinical brain metastasis samples and divided them into SEC61G high-expression and SEC61G low-expression groups. IHC staining was performed for CD206 (M2 marker), CD86 (M1 marker), and CD8 (T cell marker) (**Figure 6I**). In the SEC61G high-expression group, CD206 staining was significantly stronger, indicating a dominant M2 polarization state. In contrast, staining for CD86 and CD8 was weaker, suggesting reduced M1 polarization and T cell infiltration (**Figure 6J**). In the SEC61G low-expression group, CD206 staining was weaker, indicating reduced M2 polarization, while CD86 and CD8 staining were significantly stronger, reflecting increased M1 polarization and enhanced T cell infiltration (**Figure 6J**). These differences in staining intensity and immunohistochemical scores were statistically significant. Taken together, high SEC61G expression in clinical brain metastasis samples is associated with increased M2 polarization and reduced M1 polarization and T cell infiltration, while low SEC61G expression shows the opposite pattern.

### SEC61G expression correlates with immune evasion via M2 macrophage polarization and impaired tertiary lymphoid structures maturation in lung cancer

**Figure 7:**
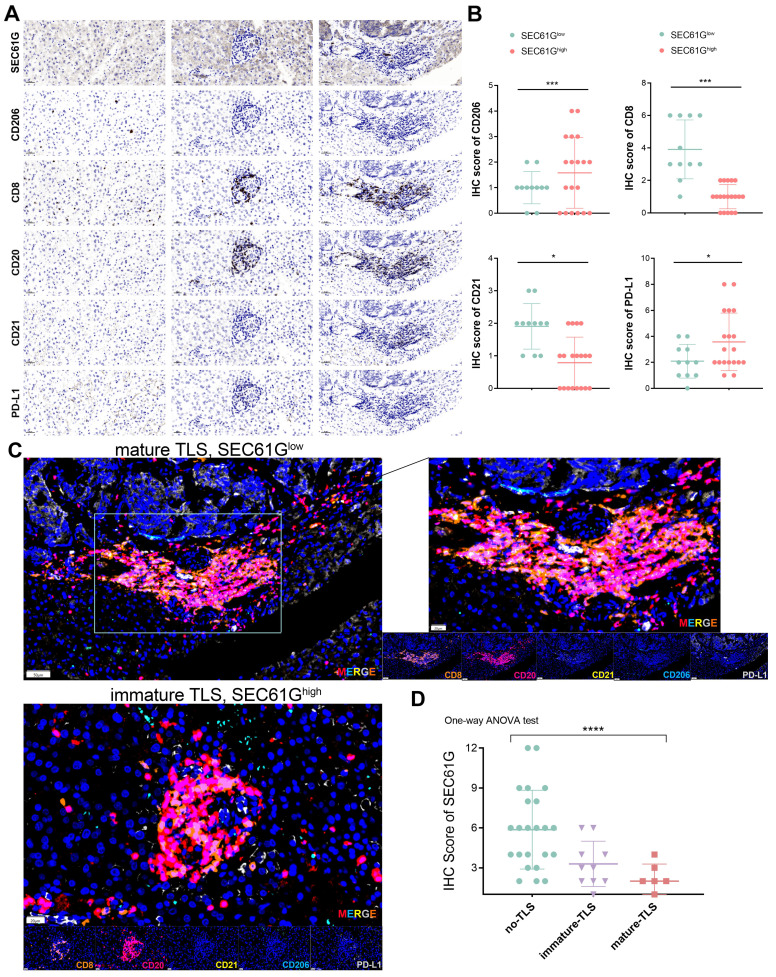
** Immune profiling of SEC61G and tertiary lymphoid structures (TLSs)-associated markers in lung cancer.** (A) Immunohistochemical staining of SEC61G, CD206, CD8, CD20, CD21, and PD-L1 in three sample groups showing differential immune cells infiltration in samples with low, moderate, and high expression of SEC61G, respectively. (B) Differential expression analysis of immune markers based on differential SEC61G expression level, with significant increases in CD206 and PD-L1 expression in the high SEC61G group, and significant decreases in CD8 and CD21 expression. (C) Multiplex IHC analysis illustrating the expression patterns of CD206, CD8, CD20, PD-L1, and CD21 in relation to TLS maturation status, revealing higher SEC61G levels in TLS-present groups and a positive correlation between CD8 infiltration and TLS maturation. (D) Boxplot representation of SEC61G expression across groups with no TLS, immature TLS, and mature TLS, indicating significantly higher expression in the no TLS group compared to TLS groups, and higher expression in the immature TLS group compared to the mature TLS group. *p < 0.05; **p < 0.01; ***p < 0.001; ****p < 0.0001.

The immune profiling of SEC61G expression revealed distinct patterns of immune cell infiltration and tertiary lymphoid structures (TLS) maturation in lung cancer samples (**Figure 7A**). Immunohistochemical staining demonstrated that high SEC61G expression was associated with increased infiltration of CD206⁺ M2 macrophages (*P* < 0.001), while CD8⁺ T cells and CD21⁺ follicular dendritic cells were markedly reduced in comparison to samples with low or moderate SEC61G expression (*P* < 0.05). Notably, PD-L1 levels significantly changed across SEC61G expression groups, suggesting SEC61G-mediated immune modulation operates in cooperation with PD-L1 (**Figure 7B**).

Differential expression analysis further confirmed that high SEC61G expression correlated with significant upregulation of CD206, a marker of immunosuppressive M2 macrophages, and downregulation of CD8 and CD21, key markers of T cell activation and TLS formation, respectively (**Figure 7C**). Multiplex IHC analysis highlighted the interplay between SEC61G expression and TLS maturation status, showing elevated SEC61G levels in TLS-present samples. Importantly, mature TLSs exhibited higher CD8⁺ T cell infiltration and CD21 expression, whereas immature TLSs and TLS-absent samples displayed significantly higher CD206 and SEC61G expression, underscoring the antagonistic relationship between SEC61G and TLS maturation (**Figure 7C**).

Boxplot analysis of SEC61G expression across TLS groups revealed a striking gradient: SEC61G expression was highest in samples lacking TLSs, intermediate in those with immature TLSs, and lowest in samples with mature TLSs (**Figure 7D**). These findings suggest that SEC61G may impede TLS maturation, thereby suppressing adaptive immune responses and facilitating immune evasion in the tumor microenvironment. Collectively, these results underscore the pivotal role of SEC61G in reshaping the immune landscape of lung cancer, particularly through its association with M2 macrophage polarization and impaired TLS maturation.

## Discussion

**Figure 8:**
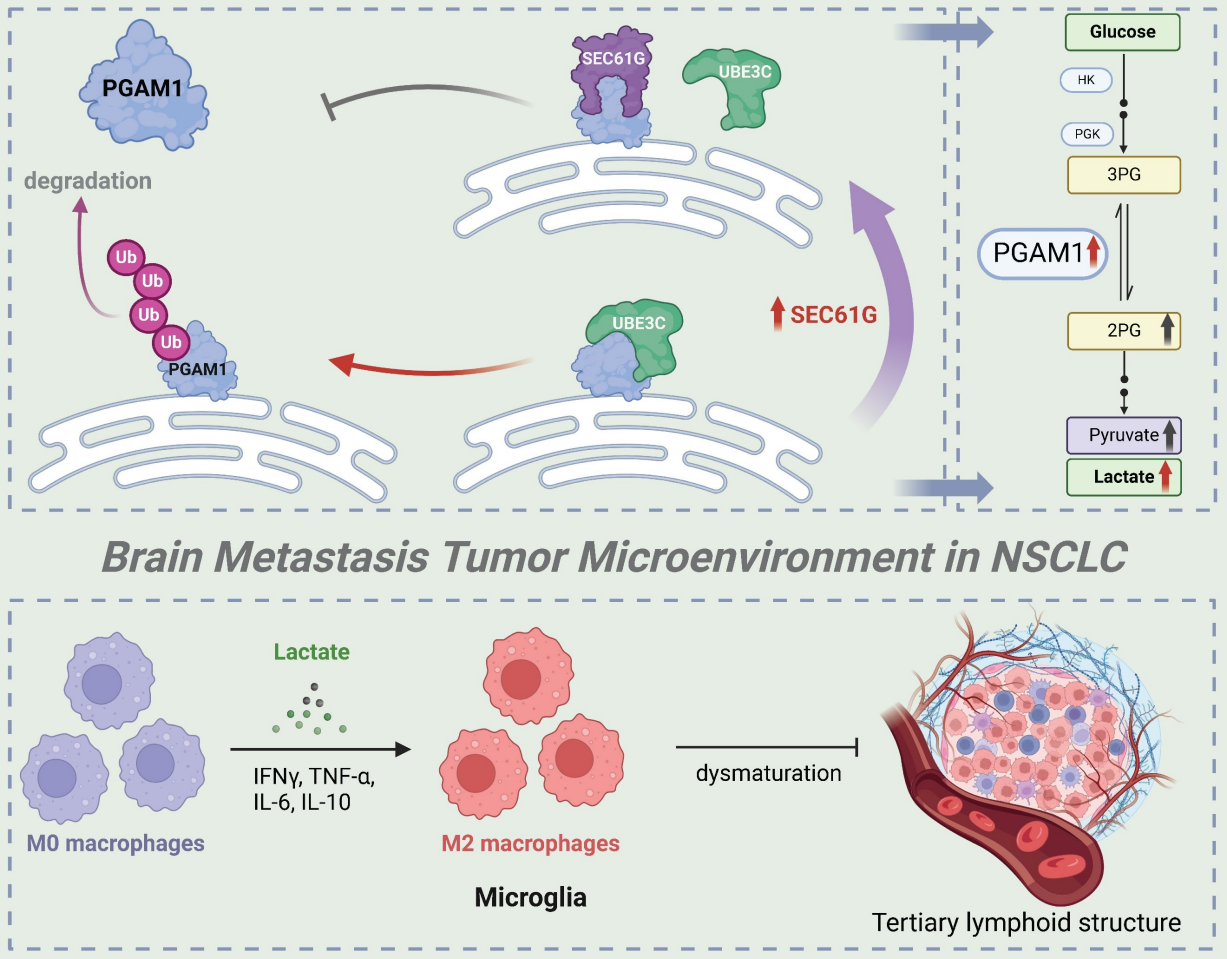
** Hypothetical model of SEC61G-mediated brain metastasis in NSCLC.** Overexpression of SEC61G in tumor cells leads to the stabilization of PGAM1, a key glycolytic enzyme, driving metabolic reprogramming towards enhanced glycolysis. This metabolic shift supports tumor cell proliferation, survival, and adaptation to the brain microenvironment. SEC61G-mediated metabolic reprogramming contributes to immune microenvironment remodeling. Elevated glycolytic activity increases lactate production, promoting the polarization of microglia to an M2-like phenotype, which facilitates immune evasion and tumor progression in the brain. SEC61G impairs maturation of TLS in the brain metastatic microenvironment, potentially suppressing anti-tumor immune responses.

This study systematically investigates the role and molecular mechanisms of SEC61G in non-small cell lung cancer (NSCLC) brain metastases, highlighting its critical role in promoting metastases through metabolic reprogramming and immune microenvironment remodeli**ng (Figure 8**). As the gamma subunit of the SEC61 translocon complex in the endoplasmic reticulum, SEC61G has been increasingly recognized for its involvement in tumorigenesis and progression. Previous studies have shown that SEC61G is highly expressed in various solid tumors, including breast cancer, glioblastoma, and renal cell carcinoma, and is closely associated with poor prognosis^6^, ^15^, ^16^. It enhances tumor cell growth and survival by regulating endoplasmic reticulum stress and protein folding homeostasis, and it promotes invasion and metastasis through activation of the EGFR signaling pathway.

Our study further reveals that SEC61G stabilizes PGAM1, a key glycolytic enzyme, by inhibiting UBE3C-mediated ubiquitination and degradation, thereby enhancing glycolytic activity. This mechanism links SEC61G to metabolic reprogramming and brain metastases. Previous studies have shown that SEC61G promotes tumor invasion through metabolic regulation in breast cancer, and its role in glycolysis is consistent with these findings^15^. Additionally, SEC61G has been implicated in metastasis of kidney cancer^16^, where it enhances tumor cells' adaptation to the brain microenvironment, further supporting its significance in NSCLC brain metastases. Given that other subunits of the SEC61 complex, such as SEC61A1, are being explored as drug targets, SEC61G may also represent a promising therapeutic target^17^. Developing SEC61G-specific inhibitors or disrupting its interaction with UBE3C could effectively suppress tumor glycolysis and prevent metastases. Moreover, as SEC61G is overexpressed across multiple cancer types, its therapeutic potential extends beyond NSCLC.

PGAM1, as a central enzyme in the glycolytic pathway, plays a key role in tumor metabolic reprogramming^33^. It is highly expressed in cancers such as breast cancer, liver cancer, and glioblastoma and is closely associated with tumor invasiveness and metastatic potential^29^. PGAM1 promotes the accumulation of glycolytic intermediates, providing energy and biosynthetic precursors for rapid tumor growth^34^. Its metabolic product, lactate, acidifies the tumor microenvironment, suppresses immune cell activity, and facilitates immune evasion^35^, ^36^. This study demonstrates that SEC61G enhances PGAM1 stability by inhibiting its ubiquitination, thereby driving glycolysis and lactate production. These findings align with existing evidence linking PGAM1 to brain metastases, where it promotes tumor colonization in the brain through the Warburg effect. Additionally, PGAM1 has been shown to regulate lactate secretion in breast cancer, inducing immunosuppressive cell activity^37^, ^38^. Combined targeting of SEC61G and PGAM1 may therefore represent an effective therapeutic strategy. SEC61G inhibitors could destabilize PGAM1, while PGAM1 inhibitors could block its enzymatic activity, jointly suppressing tumor metabolism and improving the immunosuppressive tumor microenvironment^39^.

Metabolic reprogramming is a critical mechanism by which tumor cells adapt to heterogeneous microenvironments^40^, ^41^. The Warburg effect, characterized by tumor cells favoring glycolysis for energy production even under aerobic conditions, is considered a hallmark of tumor metastasis. This study identifies that SEC61G drives glycolytic metabolism by stabilizing PGAM1, a process that not only provides sufficient energy for tumor cells but also alters the tumor microenvironment through lactate secretion. Previous studies have shown that enhanced glycolysis is a hallmark of tumor cell adaptation to the brain microenvironment^42^, ^43^. For example, in glioblastoma, glycolytic byproducts such as lactate suppress T-cell function and enhance the activity of immunosuppressive cells in the brain, thereby facilitating tumor cell colonization^44^, ^45^. Additionally, lactate has been reported to increase the permeability of the blood-brain barrier, creating a favorable condition for tumor cells to invade brain tissue. The SEC61G-UBE3C-PGAM1 axis driving glycolytic metabolic reprogramming observed in this study aligns with these findings, further emphasizing the central role of metabolic regulation in NSCLC brain metastases.

The remodeling of the immune microenvironment is a critical mechanism for immune evasion and metastasis in tumor cells^46^-^48^. Lactate has been shown to suppress the activity of effector T cells and natural killer cells while promoting the differentiation and function of regulatory T cells (Tregs) and myeloid-derived suppressor cells (MDSCs). By reducing cytotoxic T-cell infiltration and activity, lactate creates a permissive environment for tumor growth^49^, ^50^. Additionally, lactate acts as a signaling molecule to upregulate immune checkpoint molecules such as PD-L1 on tumor-associated macrophages (TAMs), further reinforcing immune evasion mechanisms. This study demonstrates that lactate produced through SEC61G-driven metabolic reprogramming induces M2 polarization of microglia in the brain. M2-polarized microglia secrete immunosuppressive factors such as IL-6 and IL-10, further suppressing antitumor immune responses and creating an immune microenvironment conducive to brain metastases^51^. Existing literature has highlighted the role of M2-polarized microglia in tumor brain metastases. For instance, in breast cancer brain metastasis models, M2-polarized microglia enhance tumor cell survival and growth^52^. These cells also promote the recruitment of Tregs, further dampening the anti-tumor immune response^32^. Moreover, lactate has been widely reported as an inducer of M2 polarization, suppressing pro-inflammatory responses of M1 microglia and creating an immunosuppressive microenvironment^53^, ^54^. Our findings are consistent with prior studies demonstrating that lactate can polarize TAMs and microglia toward an M2-like phenotype. This phenotype is characterized by enhanced anti-inflammatory and immunosuppressive activities, contributing to tumor immune evasion and resistance to therapies. The SEC61G-mediated microglial polarization observed in this study further supports this mechanism and provides new evidence in the context of NSCLC brain metastases.

This study identifies SEC61G as a pivotal regulator of immune evasion in lung cancer, primarily through promoting M2 macrophage polarization and impairing tertiary lymphoid structures (TLS) maturation. High SEC61G expression was associated with increased infiltration of immunosuppressive CD206⁺ M2 macrophages, reduced CD8⁺ T cells, and diminished CD21⁺ follicular dendritic cells, correlating with compromised adaptive immune responses. Notably, SEC61G expression showed a striking inverse correlation with TLS maturation: highest in TLS-absent samples, intermediate in immature TLS, and lowest in mature TLS, underscoring its role in disrupting immune-organizing structures. Furthermore, elevated PD-L1 levels in high-SEC61G samples suggest a cooperative mechanism in reinforcing immune suppression via both macrophage-driven immunosuppression and checkpoint inhibitor pathways^47^, ^48^, ^55^. These findings highlight SEC61G as a key driver of an immunosuppressive tumor microenvironment and a potential therapeutic target to restore TLS maturation and anti-tumor immunity in lung cancer^56^-^58^.

Despite the significant findings, this study has several limitations. The clinical sample size is relatively small, and while bioinformatic analyses and tissue validation confirm SEC61G's high expression and its association with brain metastases, larger-scale studies are needed for further validation. Additionally, the lack of specific SEC61G inhibitors limits the exploration of its therapeutic potential, as the study primarily relies on gene silencing and overexpression models. Furthermore, the complexity of the brain metastatic microenvironment, involving various cell types such as astrocytes, endothelial cells, and T cells, remains insufficiently explored. It is also unclear whether lactate contributes to microenvironment remodeling through additional unidentified pathways, warranting further investigation.

Therefore, this study reveals that SEC61G promotes NSCLC brain metastases by stabilizing PGAM1, driving glycolytic metabolic reprogramming, and remodeling the immune microenvironment through lactate production. These findings deepen our understanding of tumor metabolism and immune regulation while providing a foundation for therapeutic strategies targeting SEC61G and PGAM1. Future research should validate their roles in brain metastases across other cancer types and explore the broader metabolic and immune regulatory networks involving SEC61G and PGAM1. These efforts could pave the way for novel therapeutic approaches in managing NSCLC brain metastases.

## Conclusion

In conclusion, this study reveals that SEC61G plays a pivotal role in regulating microglial polarization and shaping the TME in lung cancer brain metastases. SEC61G promotes the K48-linked polyubiquitination and stabilization of PGAM1, enhancing glycolysis and driving the polarization of microglia towards an M2 phenotype. This is evidenced by increased expression of the M2 marker CD206 and elevated secretion of cytokines IL-6 and IL-10, while suppressing the M1 marker CD86. Inhibition of PGAM1 reverses these effects, highlighting the metabolic dependence of SEC61G-induced M2 polarization.

*In vivo* and clinical data further confirm that SEC61G expression correlates with M2 polarization and reduced anti-tumor immune activity, as seen in enhanced CD206 expression and decreased CD8^+^ T cell infiltration in high-SEC61G conditions. Conversely, SEC61G knockdown promotes M1 polarization, T cell infiltration and TLS maturation, favoring an anti-tumor immune response. In summary, SEC61G drives an immunosuppressive, tumor-promoting microenvironment in brain metastases through the PGAM1-mediated glycolytic pathway, offering a potential therapeutic target to enhance anti-tumor immunity.

## Supplementary Material

Supplementary figures and tables.
