# Supplementary material for: SEC61G Facilitates Brain Metastases via Antagonizing PGAM1 Ubiquitination and Immune Microenvironment Remodeling in Non-Small Cell Lung Cancer
Source: Int J Biol Sci. 2025 Jan 27;21(4):1436–58. doi: 10.7150/ijbs.109187 (PMC11844280; doi:10.7150/ijbs.109187)
Supplement: Supplementary file 1 — Supplementary figures and tables. [file ijbsv21p1436s1.pdf]

Supplementary Figure 1. Identification of Brain Metastasis-Associated Genes in Mouse Models

(A) Heatmap of the top 50 upregulated genes identified from differential expression analysis of the GSE131907 single-cell RNA sequencing dataset, comparing epithelial cells from brain metastases with those from normal lung and primary tumor tissues. (B) Heatmap of the top 50 differentially expressed genes ($|\log2\ FC| > 1$, $P < 0.05$) in transcriptome sequencing of the brain-metastatic H2030-BrM5 cell line compared to its parental H2030 line.

Supplementary Figure 2. SEC61G Promotes Enhances Proliferation in Lung Cancer Cells. CCK-8 assay showing that overexpression of SEC61G significantly increases the proliferation of H2030 and PC9 cells.

Supplementary Figure 3. SEC61G Expression Correlates with Ubiquitination Related Processes and Protein-Protein Interaction Networks in Lung Cancer Cells.

(A) Transcriptomic analysis of lung cancer cell lines also showed a significant inverse correlation between SEC61G expression and ubiquitination-related biological processes. (B) Protein-protein interaction networks and epigenetic regulatory analyses revealed that SEC61G, PGAM1, and UBE3C are closely linked in processes such as protein ubiquitination and tumor metabolic regulation (Supplementary Figure 2B).

Supplementary Figure 4. Identification of Potential Lysine Ubiquitination Sites on PGAM1 Through Bioinformatic Analysis.

We analyzed predicted ubiquitination sites on PGAM1 (amino acids 100-200) from the PhosphoSitePlus database, identifying five potential lysine ubiquitination sites.

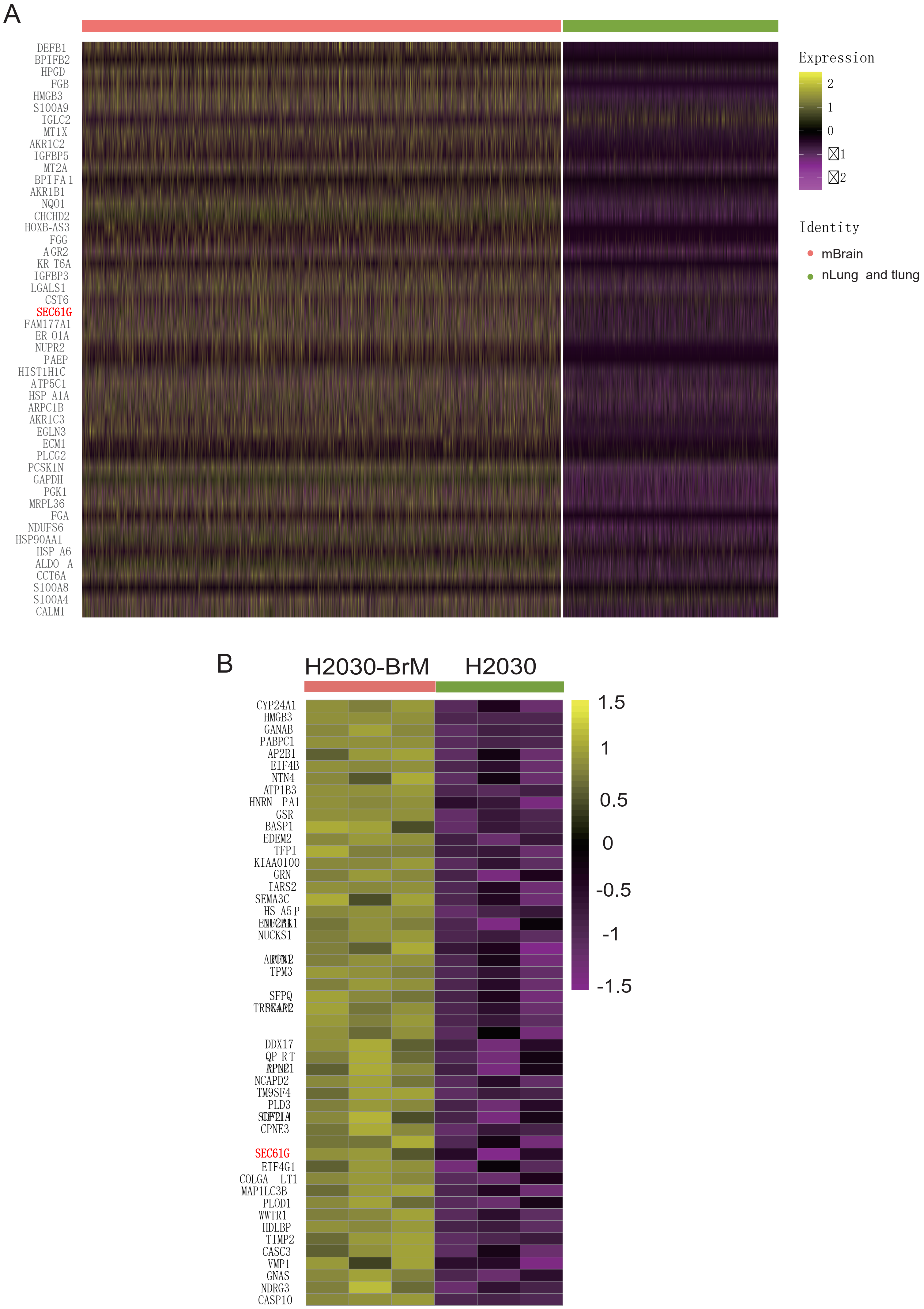
A
DEFB1
BPIFB2
HPGD
FGB
HMGB3
S100A9
IGLC2
MT1X
AKR1C2
IGFBP5
MT2A
BPIFA1
AKR1B1
NQO1
CHCHD2
HOXB-AS3
FGG
AGR2
KRT6A
IGFBP3
LGALS1
CST6
SEC61G
FAM177A1
ERO1A
NUPR2
PAEP
HIST1H1C
ATP5C1
HSPA1A
ARPC1B
AKR1C3
EGLN3
ECM1
PLCG2
PCSK1N
GAPDH
PGK1
MRPL36
FGA
NDUFS6
HSP90AA1
HSPA6
ALDOA
CCT6A
S100A8
S100A4
CALM1
Expression
2
1
0
-1
-2
Identity
mBrain
nLung and tlung
B
H2030-BrM
H2030
CYP24A1
HMGB3
GANAB
PABPC1
AP2B1
EIF4B
NTN4
ATP1B3
HNRNPA1
GSR
BASP1
EDEM2
TFPI
KIAA0100
GRN
IARS2
SEMA3C
HSPA5P
NUCKS1
TPM3
SFPQ
DDX17
RPN1
NCAPD2
TM9SF4
PLD3
CPNE3
SEC61G
EIF4G1
MAP1LC3B
PLOD1
WWTR1
HDLBP
TIMP2
CASC3
VMP1
GNAS
NDRG3
CASP10
1.5
1
0.5
0
-0.5
-1
-1.5

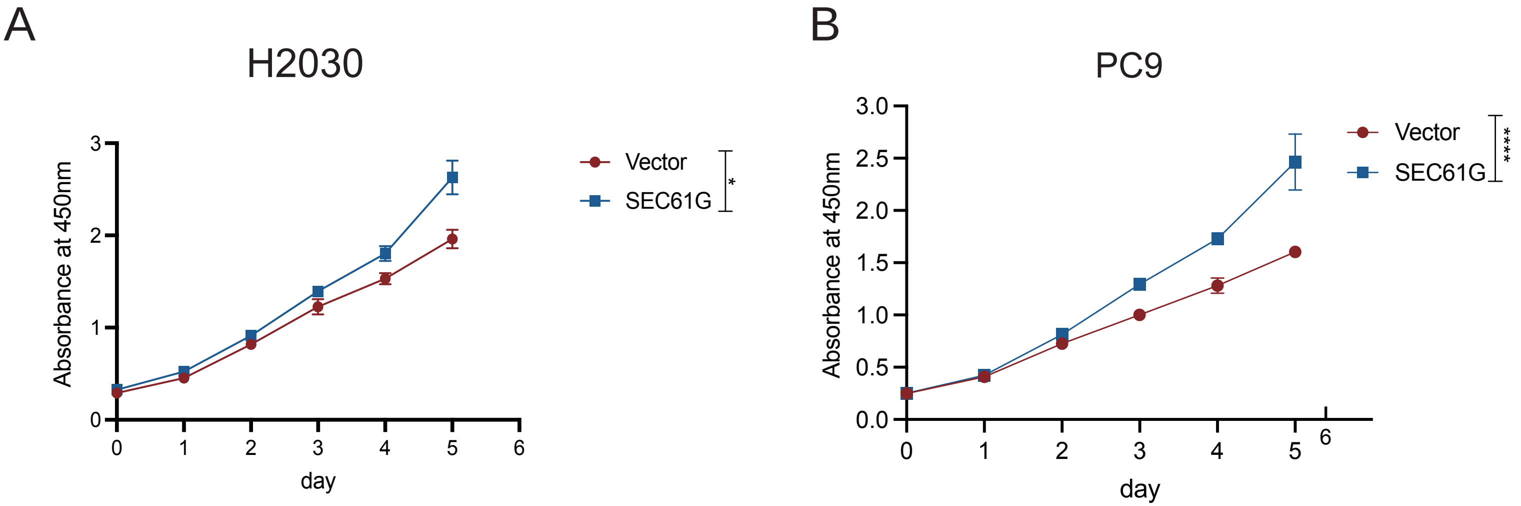
A
H2030
Absorbance at 450nm
0
1
2
3
day
0
1
2
3
4
5
6
Vector
SEC61G
*
B
PC9
Absorbance at 450nm
0.0
0.5
1.0
1.5
2.0
2.5
3.0
day
0
1
2
3
4
5
6
Vector
SEC61G
****

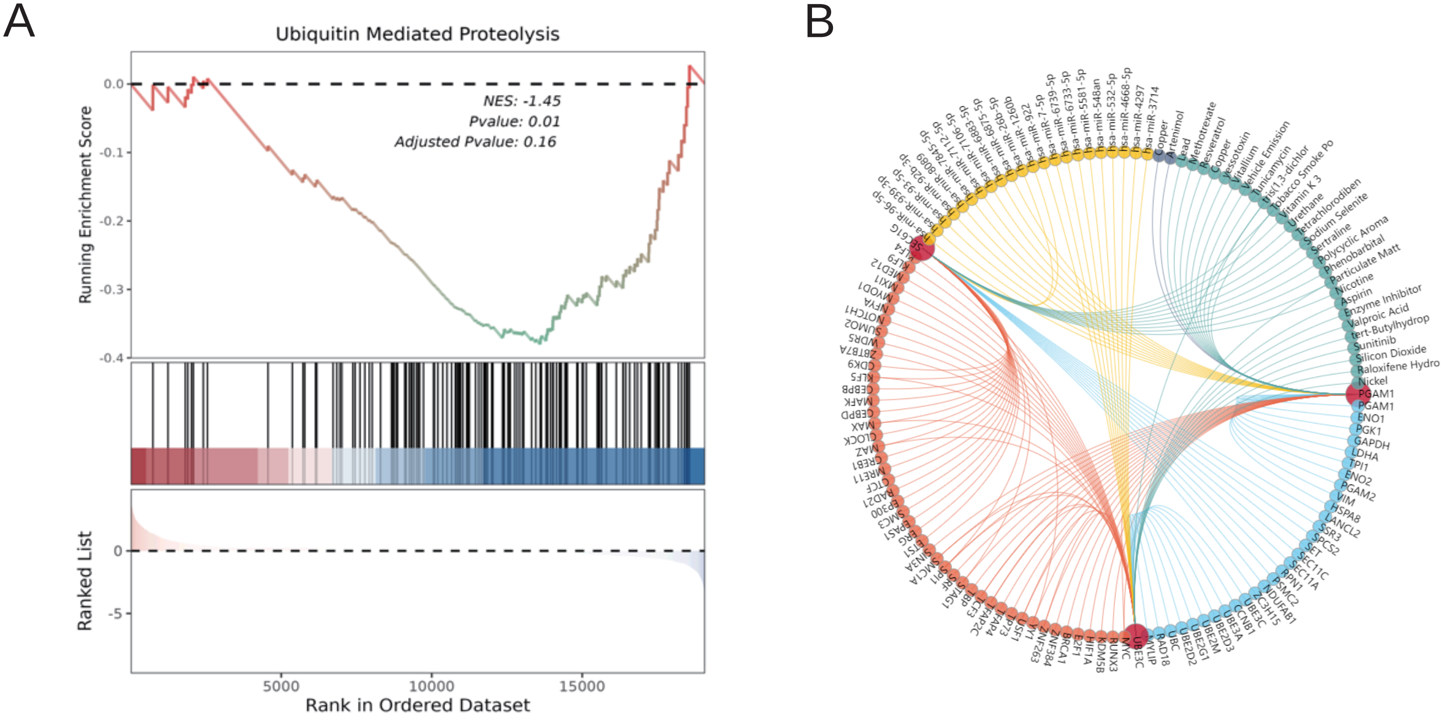
A
Ubiquitin Mediated Proteolysis
NES: -1.45
Pvalue: 0.01
Adjusted Pvalue: 0.16
Running Enrichment Score
0.0
-0.1
-0.2
-0.3
-0.4
Ranked List
0
-5
5000
10000
15000
Rank in Ordered Dataset
B
hsa-miR-4297
hsa-miR-3714
Copper
Artenimol
Lead
Methotrexate
Resveratrol
Copper
Vessotoxin
Vitallium
Vehicle Emission
Tunicamycin
bis(1,3-dichlor
Tobacco Smoke Po
Vitamin K 3
Urethane
Tetrachlorodiben
Sodium Selenite
Sertraline
Polycyclic Aroma
Phenobarbital
Particulate Matt
Nicotine
Aspirin
Enzyme Inhibitor
Valproic Acid
tert-Butylhydrop
Sunitinib
Silicon Dioxide
Raloxifene Hydro
Nickel
PGAM1
PGAM1
ENO1
PGK1
GAPDH
LDHA
TPI1
ENO2
PGAM2
VIM
HSPA8
LANCL2
SSR3
SPCS2
SEC11C
SEC11A
RPN1
PSMC2
NDUFAB1
ZC3H15
UBE3C
CCNB1
UBE3A
UBE2D3
UBE2M
UBE2G1
UBE2D2
UBC
RAD18
MYLIP
UBE3C
MYC
RUNX3
KDM5B
HIF1A
E2F1
BRCA1
ZNF384
ZNF263
YY1
USF1
TP73
TFAP4
TFAP2C
TCF3
TBP
STAG1
SREBF1
SMC1A
SIN3A
RELA
PGR
NFKB
NEAT1
ETS1
ERG
EPAS1
SMC3
EP300
RAD21
CTCF
MREI1
CREB1
MAZ
CLOCK
MAX
CEBPD
MAFK
CEBPB
KLF5
CDK9
ZBTB7A
WDR5
SUMO2
NOTCH1
NFYA
MYOD1
MXI1
MED12
KLF9
KLF4
SEL61G
SEL61G

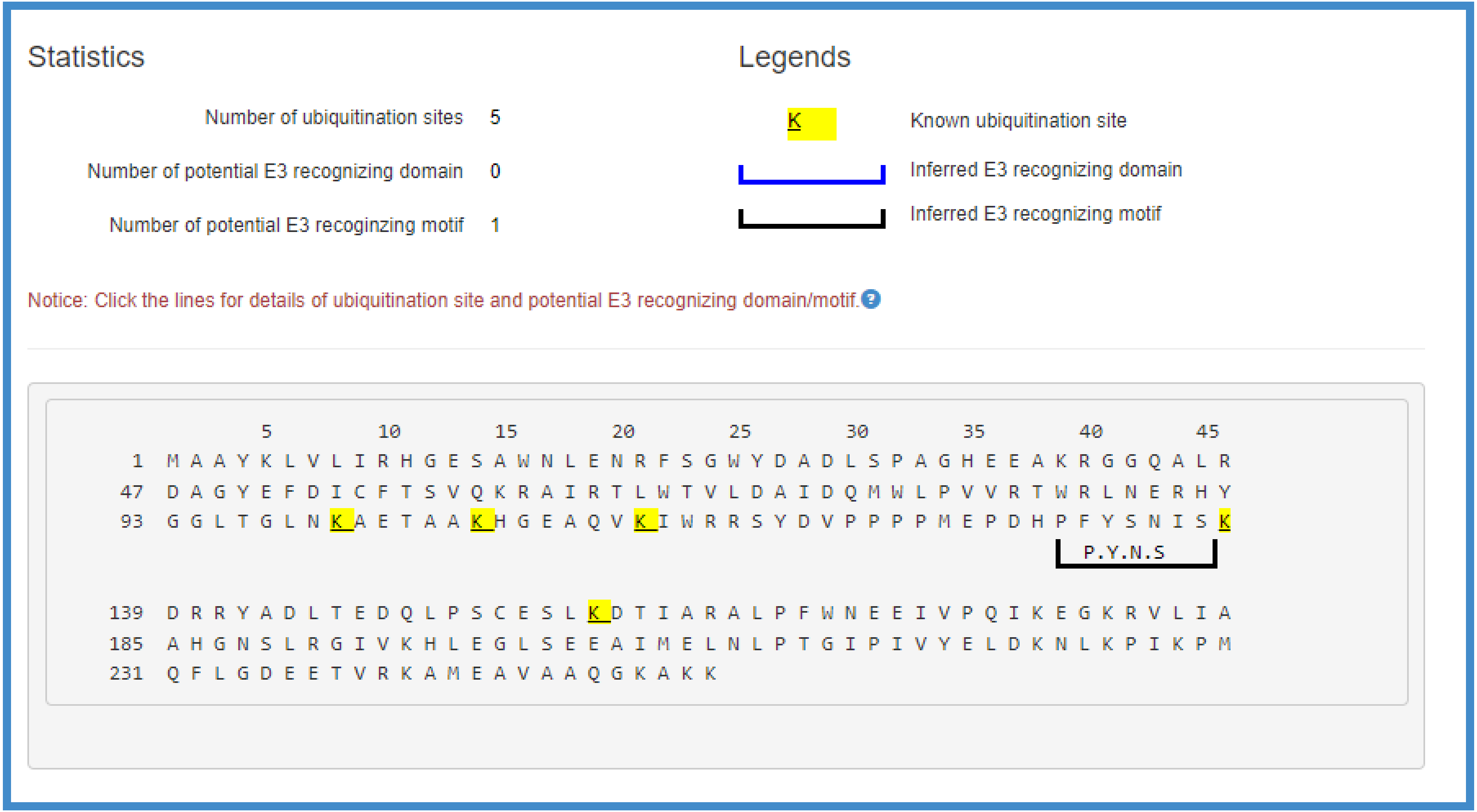
Statistics
Number of ubiquitination sites 5
Number of potential E3 recognizing domain 0
Number of potential E3 recoginzing motif 1
Legends
K
Known ubiquitination site
Inferred E3 recognizing domain
Inferred E3 recognizing motif
Notice: Click the lines for details of ubiquitination site and potential E3 recognizing domain/motif.
5 10 15 20 25 30 35 40 45
1 M A A Y K L V L I R H G E S A W N L E N R F S G W Y D A D L S P A G H E E A K R G G Q A L R
47 D A G Y E F D I C F T S V Q K R A I R T L W T V L D A I D Q M W L P V V R T W R L N E R H Y
93 G G L T G L N K A E T A A K H G E A Q V K I W R R S Y D V P P P P M E P D H P F Y S N I S K
P.Y.N.S
139 D R R Y A D L T E D Q L P S C E S L K D T I A R A L P F W N E E I V P Q I K E G K R V L I A
185 A H G N S L R G I V K H L E G L S E E A I M E L N L P T G I P I V Y E L D K N L K P I K P M
231 Q F L G D E E T V R K A M E A V A A Q G K A K K

Table S1. Primer Sequencing list.

Gene ID	Sequencing（5’3’）
PGK1-F	CAAGGTTAAAGCCGAGCCAGCCAA
PGK1-R	GCCTTCTGTGGCAGATTGACTCC
HMOX1-F	AAGACTGCGTTCCTGCTCAAC
HMOX1-R	AAAGCCCTACAGCAACTGTCG
HK2-F	GATTGTCCGTAACATTCTCATCGA
HK2-R	TGTCTTGAGCCGCTCTGAGAT
PFKL-F	CACAGGTGCCAACATCTTCCGCA
PFKL-R	TCATGTCGGTGCCGCAGAAGTCG
PGAM1-F	ATGATGTCCCACCACCTCCGAT
PGAM1-R	ATCCTTCAGACTCTCACAGGAG
ENO1-F	GCTCCGGGACAATGATAAGACTCG
ENO1-R	CTGTTCCATCCATCTCGATCATC
PKM2-F	CAAAGGACCTCAGCAGCCATGTC
PKM2-R	GGGAAGCTGGGCCAATGGTACAGA
LDHA-F	TGGAGATTCCAGTGTGCCTGTATGG
LDHA-R	CACCTCATAAGCACTCTCAACCACC
ALDOB-F	CACCATTCAAGGGCTTGATGGCCT
ALDOB-R	TTCCTGGATAGCGAGGCTGGAT
CXCL9-F	CCACCCGAACGTCTTATCTAATC
CXCL9-R	GTGGGTCACAGACTCTCAAAT
iNOS-F	GCAGAATGTGACCATCATGG
iNOS-R	ACAACCTTGGTGTTGAAGGC
IL1β-F	TGATGTTCCCATTAGACAGC
IL1β-R	GAGGTGCTGATGTACCAGTT
TNFα-F	GTAGCCCACGTCGTAGCAAA
TNFα-R	CCCTTCTCCAGCTGGGAGAC
CD86-F	TAGGGATAACCAGGCTCTAC
CD86-R	CGTGGGTGTCTTTTGCTGTA

CD206-F	GGACGTGGCTGTGGATAAAT
CD206-R	ACCCAGAAGACGCATGTAAAG
IGF1-F	CAGTTCGTGTGTGGACCAAG
IGF1-R	GTCTTGGGCATGTCAGTGTG
TGFβ1-F	TGAGTGGCTGTCTTTTGACG
TGFβ1-R	GGTTCATGTCATGGATGGTG
CCL2-F	TTCACTGGCAAGATGATCCC
CCL2-R	TGCTTGAGGTGGTTGTGGAA
CCR2-F	ATGCTGTCCACATCTCGTTCTCG
CCR2-R	TTATAAACCAGCCGAGACTTCCTGC

Table S2. Expression of SEC61G and Clinicopathological Characteristics in Lung Adenocarcinoma Tissue Microarray (n=79).

	SEC61G		P value
	High-expression（N=40）	Low-expression low（N=39）	
**Sex**			0.433
Male	22 (55.0%)	18 (46.2%).	
Female	18 (43.9%)	21 (55.3%)	
**Age**			**0.0137**
Mean ± SD	61.5 ± 9.28	55.9 ± 10.4	
**Number of lymph node metastases**			**0.0132**
Mean ± SD	2. 75 ± 3.04	1.18 ± 1.99	
**T stage**			**<0.001**
T1a	2 (5.0%)	17 (43.6%)	
T1b	7 (17.5%)	13 (33.3%)	
T1c	11 (27.5%)	6 (15.4%)	
T2a	10 (25.0%)	1 (2.6%)	
T2b	4 (10.0%)	1 (2.6%)	
T3	4 (10.0%)	1 (2.6%)	
T4	2 (5.0%)	0 (0%)	
**N stage**			0.116
N0	13 (32.5%)	23 (59.0%)	
N1	15 (37.5%)	10 (25.6%)	
N2	9 (22.5%)	5 (12.8%)	
N3	43(7.5%)	1 (2.6%)	
**M stage**			**<0.001**
M0	21 (52.5%)	35 (89.7%)	
M1	19 (47.5%)	4 (10.3%)	

Table S3. Univariate and Multivariate Cox Regression Analysis of Lung Adenocarcinoma Tissue Microarray (n=79)

Characteristic	Total	Univariate analysis		Multivariate analysis	
		P value	HR (95%CI)	P value	HR (95%CI)
**SEC61G**	79				
high	40		1.00 (Reference)		1.00 (Reference)
low	39	**0.002**	0.29 (0.13-0.63)	**0.018**	0.31 (0.12-0.82)
**T stage**					
T1a	19		1.00 (Reference)		1.00 (Reference)
T1c	20	0.165	2.17 (0.73-6.49)	0.406	0.55 (0.14-2.24)
T1b	17	0.384	1.67 (0.53-5.27)	0.226	2.24 (0.61-8.30)
T2a	5	**0.010**	4.91 (1.45-16.59)	0.927	1.08 (0.19-6.16)
T2b	11	0.894	0.86 (0.10-7.40)	0.425	0.33 (0.02-5.02)
T3	5	0.180	2.47 (0.66-9.22)	0.494	0.57 (0.11-2.87)
T4	2	**0.045**	5.45 (1.04-28.52)	0.153	0.17 (0.02-1.92)
**N stage**					
N0	36		1.00 (Reference)		1.00 (Reference)
N1	**25**	**<.001**	5.92 (2.15-16.32)	**<.001**	8.18 (2.55-26.28)
N2	**14**	**<.001**	6.98 (2.25-21.63)	0.111	2.87 (0.78-10.50)
N3	**44**	**<.001**	27.91 (20.58-36.92)	**<.001**	32.33(20.38-84.22)
**M stage**					
M0	46		1.00 (Reference)		1.00 (Reference)
M1	23	**<.001**	4.52 (2.20-9.31)	**<.001**	10.66(3.06-37.08)

Table S4. Correlation Between SEC61G Expression Levels and Clinicopathological Characteristics in TCGA LUAD

Characteristic	Low expression of	High expression of	P value
n	267	268	
**Gender, n (%)**			0.968
Female	142 (26.5%)	144 (26.9%)	
Male	125 (23.4%)	124 (23.2%)	
**Age, n (%)**			0.094
<=65	117 (22.7%)	138 (26.7%)	
>65	140 (27.1%)	121 (23.4%)	
**T stage, n (%)**			0.349
T1	97 (18.2%)	78 (14.7%)	
T2	136 (25.6%)	153 (28.8%)	
T3	23 (4.3%)	26 (4.9%)	
T4	9 (1.7%)	10 (1.9%)	
**N stage, n (%)**			**0.023**
N0	182 (35.1%)	166 (32%)	
N1-3	72 (13.9%)	99 (19.1%)	
**M stage, n (%)**			0.547
M0	172 (44.6%)	189 (49%)	
M1	14 (3.6%)	11 (2.8%)	
**Stage, n (%)**			0.144
Stage I	155 (29.4%)	139 (26.4%)	
Stage II	60 (11.4%)	63 (12.0%)	
Stage III	33 (6.3%)	51 (9.7%)	
Stage IV	15 (2.8%)	11 (2.1%)	
**OS event, n (%)**			**< 0.001**
Alive	196 (36.6%)	147 (27.5%)	
Dead	71 (13.3%)	121 (22.6%)	
**DSS event, n (%)**			**0.006**
Alive	205 (41.1%)	174 (34.9%)	
Dead	47 (9.4%)	73 (14.6%)	
**PFI event, n (%)**			**0.007**
Alive	170 (31.8%)	139 (26.0%)	
Dead	97 (18.1%)	129 (24.1%)	

Table S5. Univariate and Multivariate Cox Regression Analysis in TCGA-LUAD

Characteristics	Total(n)	Univariate analysis		Multivariate analysis	
		Hazard ratio (95%	P value	Hazard ratio (95%	P value
**T1 vs T2**	282	1.521 (1.068-2.166)	**0.020**	1.521	0.069
**T1 vs T3&T4**	66	3.066 (1.950-4.823)	**<0.001**	2.574	**0.001**
**N0 vs N1**	94	2.382 (1.695-3.346)	**<0.001**	1.983	**<0.001**
**N0 vs N2&N3**	73	2.968 (2.040-4.318)	**<0.001**	2.437	**<0.001**
**M0 vs M1**	25	2.136 (1.248-3.653)	**0.006**	1.662	0.089
**SEC61G**	526	1.561 (1.295-1.881)	**<0.001**	1.338	**0.018**
